# Effect of Ion Concentration Changes in the Limited Extracellular Spaces on Sarcolemmal Ion Transport and Ca^2+^ Turnover in a Model of Human Ventricular Cardiomyocyte

**DOI:** 10.3390/ijms141224271

**Published:** 2013-12-13

**Authors:** Dana Hrabcová, Michal Pásek, Jiří Šimurda, Georges Christé

**Affiliations:** 1 Department of Physiology, Faculty of Medicine, Masaryk University, Kamenice 5, Brno 62500, Czech Republic; E-Mails: hrabcova.dana@email.cz (D.H.); simurda@med.muni.cz (J.Š.); 2 Institute of Thermomechanics—Branch Brno, Academy of Science of the Czech Republic, Technická 2, Brno 61669, Czech Republic; 3 EA4612 Neurocardiology, University Claude Bernard Lyon 1, 8 Ave Rockefeller, Lyon 69373 Cedex 08, France; E-Mail: georges.christe@inserm.fr

**Keywords:** human heart, cardiac cell, t-tubule, intercellular clefts, calcium, ion transport, computer model

## Abstract

We have developed a computer model of human cardiac ventricular myocyte (CVM), including t-tubular and cleft spaces with the aim of evaluating the impact of accumulation-depletion of ions in restricted extracellular spaces on transmembrane ion transport and ionic homeostasis in human CVM. The model was based on available data from human CVMs. Under steady state, the effect of ion concentration changes in extracellular spaces on [Ca^2+^]_i_-transient was explored as a function of critical fractions of ion transporters in t-tubular membrane (not documented for human CVM). Depletion of Ca^2+^ and accumulation of K^+^ occurring in extracellular spaces slightly affected the transmembrane Ca^2+^ flux, but not the action potential duration (APD_90_). The [Ca^2+^]_i_-transient was reduced (by 2%–9%), depending on the stimulation frequency, the rate of ion exchange between t-tubules and clefts and fractions of ion-transfer proteins in the t-tubular membrane. Under non-steady state, the responses of the model to changes of stimulation frequency were analyzed. A sudden increase of frequency (1–2.5 Hz) caused a temporal decrease of [Ca^2+^] in both extracellular spaces, a reduction of [Ca^2+^]_i_-transient (by 15%) and APD_90_ (by 13 ms). The results reveal different effects of activity-related ion concentration changes in human cardiac t-tubules (steady-state effects) and intercellular clefts (transient effects) in the modulation of membrane ion transport and Ca^2+^ turnover.

## 1. Introduction

The increased availability of experimental data obtained from human isolated cardiomyocytes in recent years provided sufficient material to mathematically describe the transmembrane ion transport and electrical activity in human atrial [1,2] and ventricular cells [3–8]. None of these models, however, included a description of the effects of simultaneous changes of ion concentrations in the intercellular cleft space and t-tubular system as functional compartments that may affect cellular activity. The present work is the first attempting at filling this blank.

The first model of cardiac cells that included changes of Ca^2+^ concentration in the intercellular clefts was developed by Hilgemann and Noble [9]. Focused on the description of Ca^2+^ movements and excitation-contraction (EC) coupling in rabbit atrial cells, the model accurately reproduced the variations of extracellular Ca^2+^ concentration measured in rabbit atrium [10,11]. Later, intercellular cleft space was also introduced into a model of human atrial cell by Nygren *et al.* [2].

In 2003, we published a pioneering work [12] indicating that activity-induced ion concentration changes in t-tubules of cardiac ventricular cells may be sufficiently high to modulate membrane currents and cellular electrical activity. Later, works on species-specific models for rat [13] and guinea-pig [14] demonstrated that, during a single beat at steady-state stimulation, the transient Ca^2+^ depletion in t-tubules may range between 7% and 20%, while transient K^+^ accumulation may reach 4%. This depended on the characteristics of the ion transporters, on their distribution between the surface and the t-tubular membrane, on the rate of ion diffusion between the t-tubular lumen and the extracellular space and on stimulation frequency. The resulting effect was a decrease of transmembrane Ca^2+^ influx and a reduction of sarcoplasmic reticulum (SR) Ca^2+^ load and intracellular Ca^2+^-transient. The frequency-dependent profiles of ion concentration transients during a cycle were different between these two models, stressing the need for a dedicated human ventricular cell model.

The present model of human ventricular myocyte has been formulated to take into account the up-to-date knowledge of membrane ionic currents and intracellular homeostasis in human cells. The description of the t-tubular system is based on current knowledge of morphological features in human cardiomyocytes [15]. To increase the accuracy of the model, the concentration changes in the layer adjacent to the tubular membrane were introduced similarly as in our latest work [16]. The t-tubular space was partitioned into concentric subspaces to describe the radial concentration gradients within the t-tubular lumen. In addition, to be consistent with the presence of the limited size of the pericellular interstitial space, a cleft space has been formulated, as done by Nygren *et al.* [2].

The aim of this study was to explore the functional significance of the presence of these extracellular spaces. Because the values of some parameters that are related to the features of t-tubules in human ventricular myocytes are still unknown (fractions of ionic currents in the t-tubular membrane, the rate of ion exchange between the t-tubular and extracellular space), we explored the physiological consequences of their various combinations, in particular those related to cellular electro-mechanical activity.

## 2. Results

### 2.1. Basic Behavior of the Model

**Figure 1.**
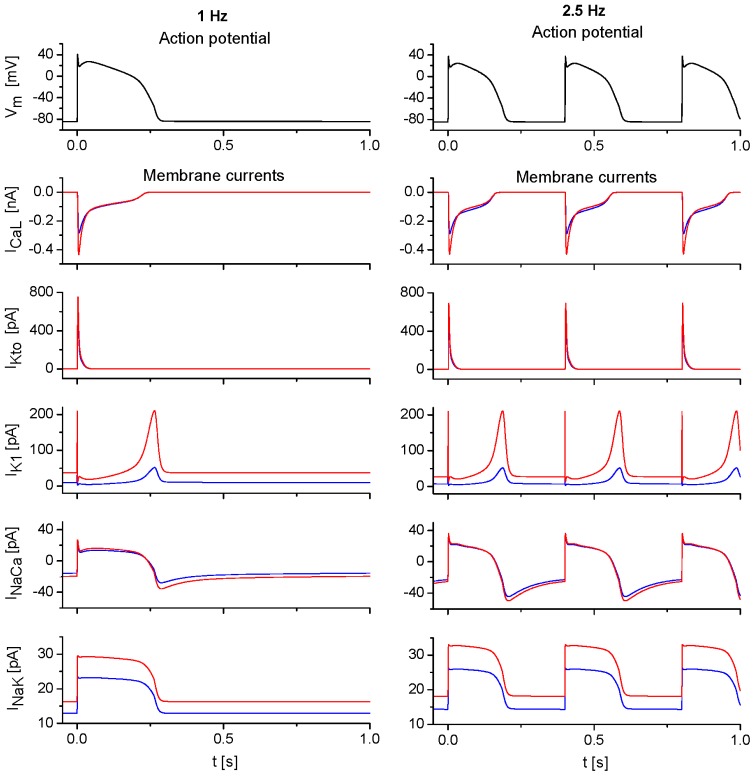
Action potentials (*V*_m_) and selected membrane currents (*I*_CaL_, *I*_Kto_, *I*_K1_, *I*_NaCa_, *I*_NaK_) at steady-state under 1 Hz (**left panel**) and 2.5 Hz (**right panel**) stimulation. Blue and red continuous lines refer to surface and t-tubular membrane, respectively.

**Figure 2.**
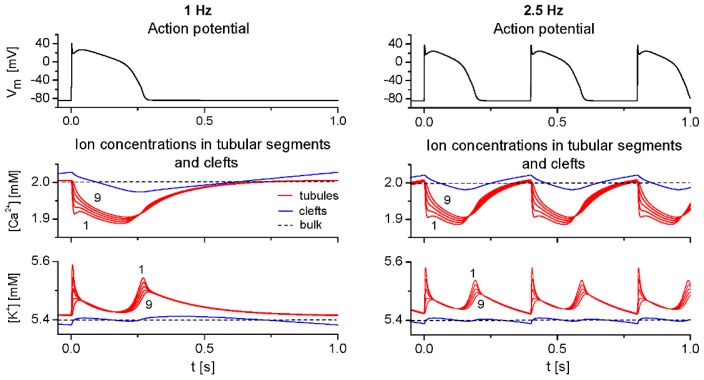
Action potentials (*V*_m_) and ion concentrations ([Ca^2+^], [K^+^]) in the first, third, fifth, seventh and ninth segments of tubular lumen (red lines) and in cleft space (blue lines) at 1 Hz (**left panel**) and 2.5 Hz (**right panel**) steady-state stimulation. Horizontal dashed lines show the levels of ion concentrations in the external bulk space.

Figure 1 illustrates the APs, three dominant ionic currents involved in membrane repolarization (*I*_CaL_, *I*_Kto_ and *I*_K1_) and currents controlling intracellular ionic homeostasis (*I*_NaCa_, *I*_NaK_) at 1 and 2.5 Hz steady-state stimulation. While the differences between APs at surface and t-tubular membranes are minimal [17], the differences between surface and tubular components of ionic currents are marked. They are caused by uneven distribution of corresponding ion transporters between both membrane systems (see Table S1 in the Supplementary Information), but also partly by differences in extracellular ion concentrations, to which both membranes are exposed during the stimulation cycle (Figure 2).

The depletion of t-tubular Ca^2+^ is predominantly induced by the activation of tubular *I*_CaL_ (*I*_CaL,t_). The two peaks in the tubular K^+^ accumulation result from the activation of tubular *I*_Kto_ (*I*_Kto,t_) at the beginning of the AP and from the increase of tubular *I*_K1_ (*I*_K1,t_) during the course of AP repolarization. The traces of tubular [Ca^2+^] and [K^+^] in Figure 2 show how activity-induced depletion of tubular Ca^2+^ and accumulation of tubular K^+^ decrease from the layer adjacent to the t-tubular membrane (first segment; [Ca^2+^]_t1_, [K^+^]_t1_) to the central part of the t-tubule lumen. Their differences demonstrate luminal ion concentration gradients in the radial direction. The alterations of [Ca^2+^] in clefts ([Ca^2+^]_c_) were induced by the activation of the main Ca^2+^ currents in the surface membrane (*I*_CaL,s_, *I*_pCa,s_, *I*_NaCa,s_) and by Ca^2+^ diffusion between t-tubules and clefts. Small changes of [K^+^]_c_ were induced by activation of *I*_Kto,s_, *I*_K1,s_ and *I*_NaK,s_ and by K^+^ diffusion between t-tubules and clefts. In addition, concentrations of all ions in the cleft space were affected by diffusion of ions between the cleft space and the external bulk space.

### 2.2. Effect of Ion Concentration Changes in Extracellular Spaces on Intracellular Ca^2+^ SR Load and Ca^2+^-Transient at Steady-State Stimulation

**Figure 3.**
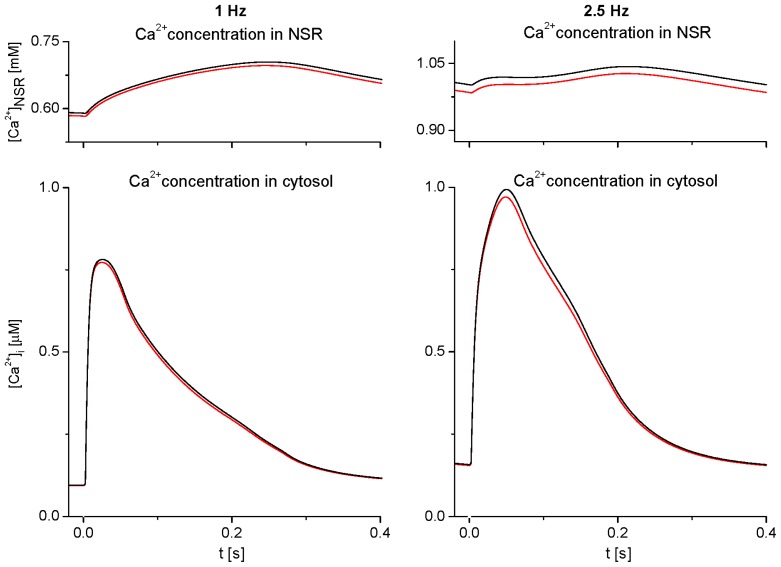
Effect of Ca^2+^ and K^+^ concentration changes in the first t-tubular segment and cleft space on Ca^2+^ concentration in the network compartment of SR ([Ca^2+^]_NSR_) and Ca^2+^-transient in the cytosol ([Ca^2+^]_i_) during a steady-state stimulation at 1 Hz (**left panel**) and 2.5 Hz (**right panel**). The results hold for basic adjustment of tubular fractions of Ca^2+^ transporters in the model cell (*f*_CaL,t_ = 0.64, *f*_NaCa,t_ = 0.56, and *f*_pCa,t_ = 0.2). The model was run when ion concentration changes in extracellular spaces were either allowed to change (red lines) or fixed at external bulk levels (black lines).

Figure 3 shows the effect of ion concentration changes in extracellular tubular and cleft spaces ([Ca^2+^]_t1_, [Ca^2+^]_c_, [K^+^]_t1_, [K^+^]_c_) described in Figure 2 on the level of Ca^2+^ load in NSR ([Ca^2+^]_NSR_) and on the intracellular Ca^2+^-transient during a steady-state stimulation cycle at 1 and 2.5 Hz. The differences between traces representing NSR Ca^2+^ load and cytosolic [Ca^2+^]-transient in the basic model before (red lines) and after fixing all extracellular ionic concentrations at external bulk values (black lines) reflect a cumulative effect of small changes in sarcolemmal Ca^2+^ transport [a decrease of Ca^2+^ entry through *I*_CaL_ and the positive part of *I*_NaCa_ by 4.7 amol/cycle (2.1%) and by 6.6 amol/cycle (2.2%) at 1 and 2.5 Hz, respectively] that are induced by variations of extracellular ion concentrations.

Though small, this effect shows a potential of these variations to slightly affect the Ca^2+^ turnover in cardiac cells at steady state. The time integral of intracellular Ca^2+^-transients was reduced by 2.5% and 3.4% at 1 and 2.5 Hz, respectively. A deeper analysis also showed that this effect is predominantly induced by depletions of Ca^2+^ concentration in the vicinity of the t-tubular membrane ([Ca^2+^]_t1_); the relative contribution of changes of [Ca^2+^]_c_, [K^+^]_c_ and [K^+^]_t1_ in total was below 0.3%.

**Figure 4.**
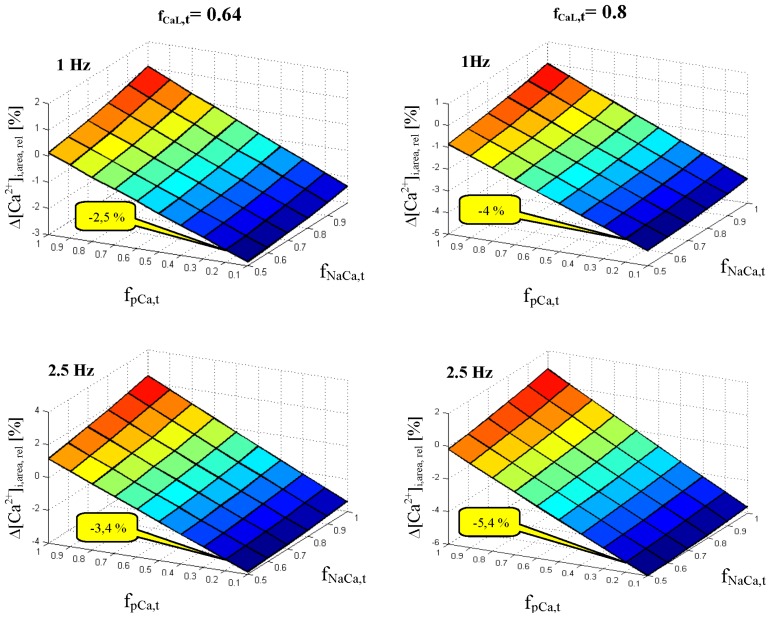
Effect of values of *f*_pCa,t_ and *f*_NaCa,t_ on intracellular Ca^2+^-transient during a steady-state cycle at *f*_CaL,t_ of 0.64 (**left panels**) and 0.8 (**right panels**). The upper and bottom panels, respectively, show the results obtained at stimulation frequency of 1 and 2.5 Hz. The values in the yellow bubble indicate the percentage of the reduction of the cytosolic Ca^2+^-transient for basic adjustment of *f*_NaCa,t_ and *f*_pCa,t_ (0.56 and 0.2, respectively). Δ[Ca^2+^]_i,area,rel_ represents the percentage change of the area delimited by cytosolic Ca^2+^-transient during a whole cycle relative to the area of steady-state Ca^2+^-transient in the model with extracellular ion concentrations fixed at external bulk levels.

To explore the effect of Ca^2+^ concentration changes in t-tubules on cytosolic Ca^2+^-transient for potentially different combinations of critical fractions *f*_CaL,t_, *f*_NaCa,t_ and *f*_pCa,t_ in human ventricular cells, we performed a series of simulations enabling us to construct these relations in 3D graphs (Figure 4). The results displayed for t-tubular fraction of L-type Ca^2+^ channels of 0.64 {based on data from guinea-pig [18] (basic setting)} showed that, if the fraction, *f*_pCa,t_, was small (<0.2), the repeated depletions of Ca^2+^ in human t-tubules might result in a reduction of Ca^2+^-transient by 3% at resting heart rate (1 Hz) and by 4% during exercise (2.5 Hz) (Figure 4, left panel). However, increasing the t-tubular fraction of L-type Ca^2+^ channels to 0.8 (consistent with data from rat cardiomyocytes [19]) would lead to a reduction of Ca^2+^-transient by more than 4% and 5.4%, respectively, under the same conditions (Figure 4, right panel). If, in addition, the rate of Ca^2+^ diffusion between human t-tubules and the cleft space was made slower (τ_Ca,ct_ increased from the basic value of 240 to 480 ms), this effect would further increase to more than 9% at 2.5 Hz (not illustrated). While the dependency of this effect on the value of *f*_NaCa,t_ appeared to be small, high values of *f*_pCa,t_ would even reverse it, as they would lead to accumulation of Ca^2+^ in the tubular space and, consequently, to a slight increase of the Ca^2+^-transient.

### 2.3. Model Responses to Sudden Changes of Stimulation Rate

**Figure 5.**
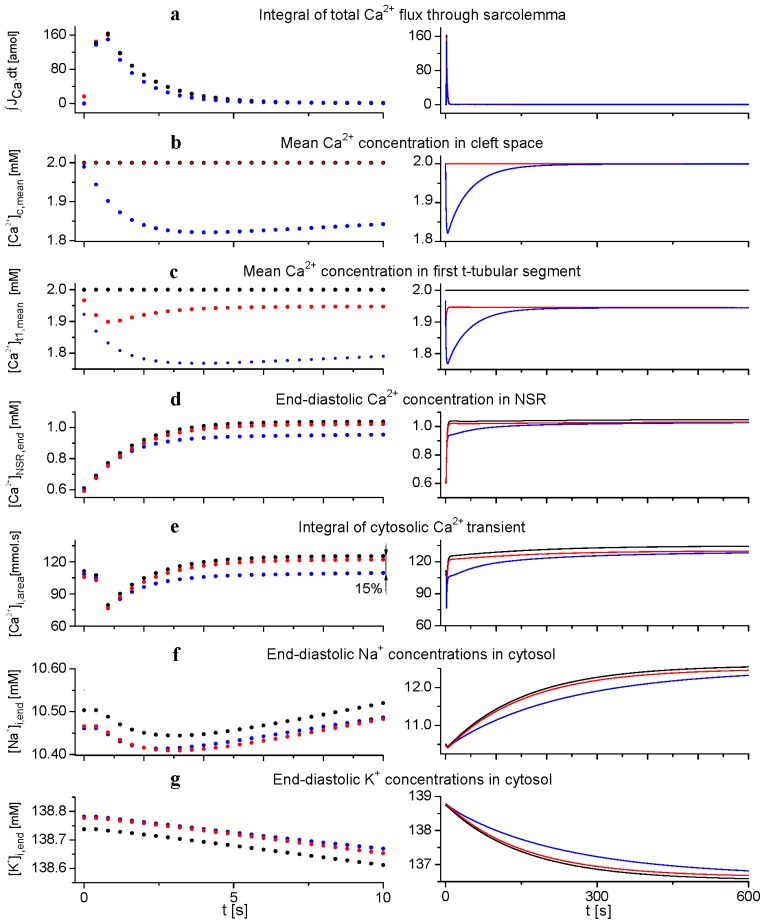
Effect of ion concentration changes in the cleft space (**b**) and in the first segment of t-tubular space (**c**) on intracellular end-diastolic ion concentrations (**d**,**f**,**g**) and on cytosolic Ca^2+^-transient (**e**, integral of cytosolic Ca^2+^-transient during a cycle) after a sudden increase of stimulation frequency from 1 (steady state, *t* = 0 s) to 2.5 Hz. The upper panel (**a**) shows the integral of total trans-sarcolemmal Ca^2+^ flux during a cycle. The left and right columns show the responses of the model during 10 and 600 s, respectively, after the change of stimulation rate. The model was run when t-tubular, and cleft ion concentrations were allowed to change (blue), when ion concentrations in the cleft space were fixed (red) and when both t-tubular and cleft ion concentrations were fixed at external bulk levels (black).

Figure 5 shows the responses of the model to a sudden change of stimulation frequency from 1 to 2.5 Hz followed during 10 s (left panels) and 600 s (right panels). It demonstrates the potency of ion concentration changes in both extracellular spaces to modulate NSR Ca^2+^ load and intracellular [Ca^2+^], [Na^+^] and [K^+^] during transient events after a sudden change of stimulation frequency. The results from the basic model (blue dots) are compared with the results after fixing ion concentrations in the cleft space (red dots) or in both t-tubular and cleft spaces (black dots) at external bulk levels. The right panels illustrate progression toward the steady state in an expanded time scale.

The fast transient increase of Ca^2+^ flux into the cell decaying within several seconds (integral of total trans-sarcolemmal Ca^2+^ flux during a cycle; panel a, left) induced a marked transient reduction of mean Ca^2+^ concentration in the cleft space (panel b, left) that was also reflected by a similar change in the mean Ca^2+^ concentration in the first tubular segment (panel c, left). Note that this reduction of [Ca^2+^] in both extracellular spaces was substantially larger (blue dots) than the reduction of [Ca^2+^]_t1_ in the model after fixing ion concentrations in clefts space (red dots). This initial rapid reduction in extracellular [Ca^2+^] decays with a time constant of approximately 50 s due to equilibration between the clefts and bulk space (panels b, right, and c, right).

The consequence of such a profound transient depletion of Ca^2+^ in both cleft and t-tubular spaces was a parallel decrease of Ca^2+^ concentration in NSR and a significant reduction of cytosolic Ca^2+^-transient by 15% at 10 s after the onset of faster stimulation (panels d, left, and e, left). This deflection decays towards steady-state values in parallel with [Ca^2+^] in cleft space (panels d, right, and e, right). The slow cumulative changes in [Na^+^]_i_ and [K^+^]_i_ (time constant of about 250 s) related to sodium and potassium currents, Na/Ca exchange and Na/K ATPase activity are also affected by changes in extracellular Ca^2+^ concentrations (panels f and g). The underlying processes lead to the final steady state in which the net total transmembrane Ca^2+^ transport (across the surface and tubular membrane) equals zero in the course of each stimulation period [see Condition (1) in the Discussion].

**Figure 6.**
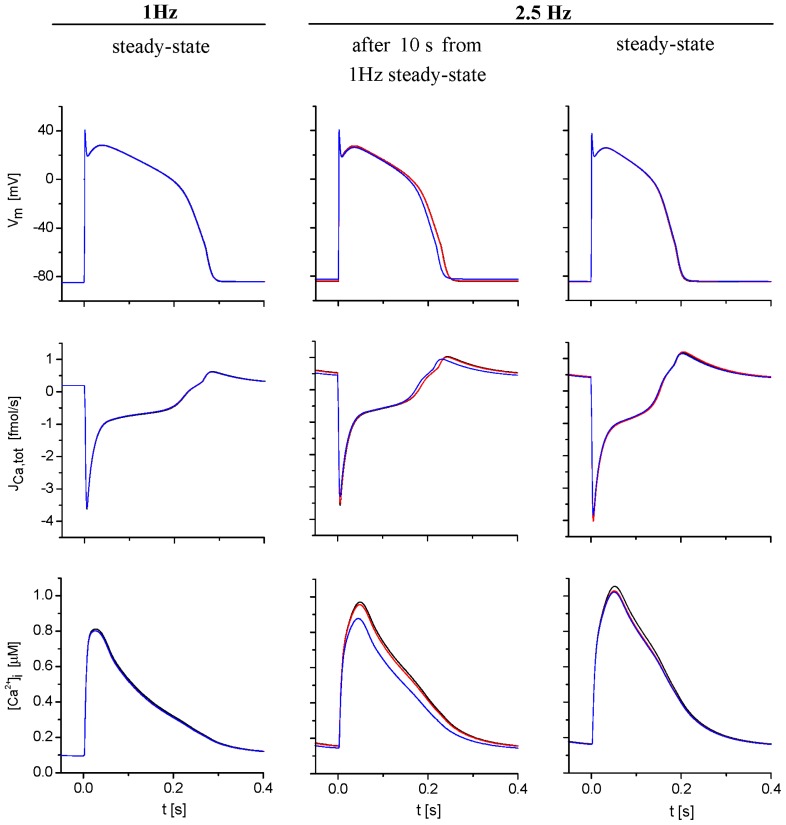
The effect of ion concentration changes in extracellular spaces on the action potential (AP) (*V*_m_), total sarcolemmal Ca^2+^ flux (*J*_Ca,tot_) and cytosolic Ca^2+^-transient after a sudden increase of stimulation frequency from 1 to 2.5 Hz. The left, middle and right columns show the results obtained, respectively, at 1 Hz steady-state stimulation, at 10 and at 600 s (steady state) after a sudden increase of simulation rate to 2.5 Hz. The model was run when t-tubular and cleft ion concentrations were allowed to change (blue lines), when ion concentrations in the cleft space were fixed (red lines) and when both t-tubular and cleft ion concentrations were fixed at external bulk levels (black, practically identical to red traces).

These results suggest that, while the role of ion concentration changes in cleft spaces in modulating cellular electrical activity and the Ca^2+^-transient is relatively small at steady state, it becomes important during perturbations, due to a change in stimulation frequency. This is illustrated in Figure 6 showing APs, Ca^2+^ flow through sarcolemma and [Ca^2+^]_i_-transient at a stimulation frequency of 1 and 2.5 Hz under conditions of free running and fixed extracellular concentrations. Steady-state differences in the course of AP were negligible, and changes in [Ca^2+^]_i_-transient were small at any frequency. In contrast, both quantities were markedly affected 10 s after a sudden change of stimulation rate from 1 to 2.5 Hz. APD_90_ (the duration of the action potential at 90% repolarization) was reduced by 13 ms in accordance with a decreased influx of Ca^2+^ ions in the course of AP. [Ca^2+^]_i_-transient dropped by 15%. If the stimulation frequency returned from 2.5 to 1 Hz, the opposite deviations in AP and [Ca^2+^]_i_-transient appeared (not shown).

To verify the ability of our model to reproduce the changes in [K^+^] and [Ca^2+^] in the clefts that were documented in the literature, we simulated, from steady-state stimulation at 1 Hz, the application of a train of stimuli at 3 Hz for durations from 34 up to 700 s and a subsequent return to 1 Hz for 700 s.

**Figure 7.**
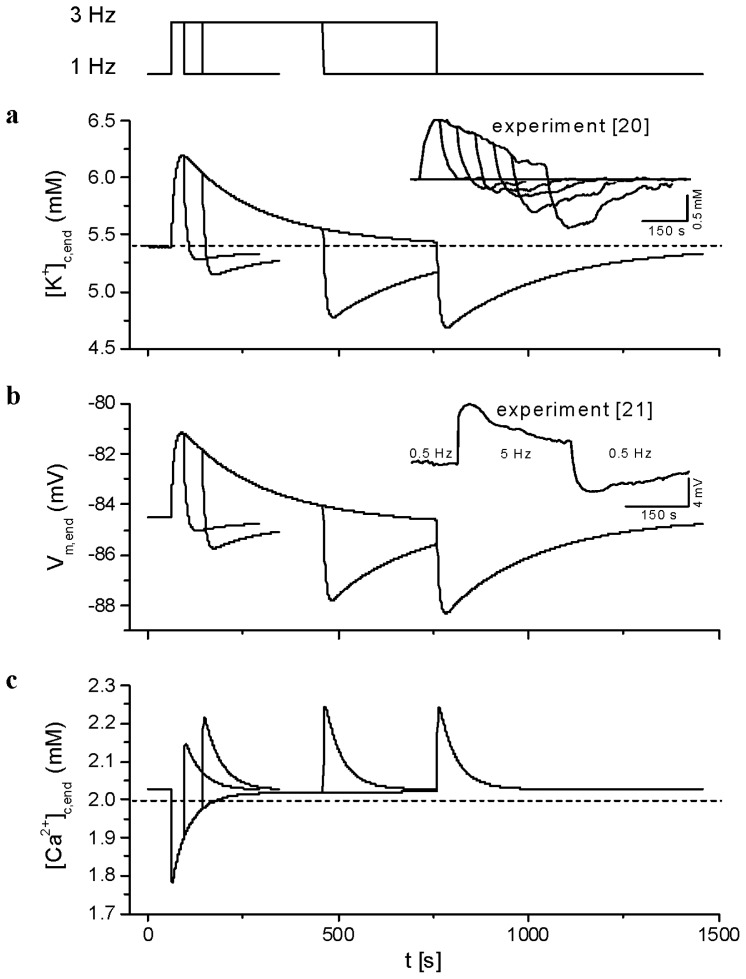
Accumulation-depletion phenomena during and after a high frequency train of stimulations. From a period of stimulation at 1 Hz, a train of stimuli at 3 Hz has been applied for four different durations: 34, 144, 400 and 700 s. From up to down are plotted superimposed outputs of the model for the four durations: the timing of changes in the frequency of stimulation, the time course of changes in the end-diastolic values of cleft K^+^ concentration (**a**), membrane voltage (**b**) and cleft Ca^2+^ concentration (**c**). The horizontal dashed lines show the levels of corresponding ion concentrations in external bulk solution. The insets show the corresponding experimental records digitized from Kunze [20] and Attwell *et al.* [21].

Figure 7 shows the end-diastolic values of K^+^ and Ca^2+^ concentrations in the cleft space ([K^+^]_c,end_ and [Ca^2+^]_c,end_) and the end-diastolic values of resting transmembrane voltage (*V*_m,end_) at the peripheral membrane. During the train of stimuli applied at 3 Hz, K^+^ accumulation was taking place initially with a time constant of 10 s and peaked at 0.8 mM above the initial level of 5.41 mM at 1 Hz. While the stimulation train was continued, it thereafter decayed with a time constant of 214 s toward the initial level at 1 Hz. A similar time course was observed for the K^+^ depletion upon returning to 1 Hz stimulation. In addition, the peak level of [K^+^]_c,end_ during the depletion depended on the duration of the stimulation train at high frequency.

These features compare well with the measurements of interstitial [K^+^] done with a K^+^-selective microelectrode by Kunze [20]. Another feature of Kunze’s data that was well reproduced in our model is that the peak magnitude of the K^+^ depletion upon returning to 1 Hz depended on the duration of the stimulation at 3 Hz.

Changes in the membrane voltage closely paralleled the kinetics of [K^+^] changes in the clefts: the membrane was depolarized during K^+^ accumulation and hyperpolarized during K^+^ depletion, by about 4 mV. The onset of the depolarization at the application of 3 Hz stimulation and that of the hyperpolarization upon returning to 1 Hz stimulation were as fast as from the microelectrode recordings of Attwell *et al.* [21]. After the peak, the time courses of the subsequent decay of the depolarization or of the hyperpolarization were comparable to those of the membrane voltage signal in [21] and of the [K^+^] signal in [20]. Thus, the cumulative K^+^ accumulation and membrane depolarization measured at the end of diastole in the model during a high frequency stimulation train are comparable to published data, given the differences with our modelling: (i) unlike Kunze’s and Attwell *et al.*’s data, which were recorded in the atrial tissues of rabbit and guinea-pig, respectively, our model is designed for human ventricular cells; and (ii) the tissues were not perfused, whereas our model assumes an infinite volume of the bulk solution, which is equivalent to a perfect perfusion.

In our simulations, the reduction of [Ca^2+^]_c,end_ at the beginning of the 3 Hz stimulation train was fast and amounted to 0.25 mM (Figure 7). Subsequently, cleft [Ca^2+^] decayed with a time constant of 46 s, while the model cell was still stimulated at 3 Hz. In addition, our simulations show that, upon returning to 1 Hz, a transient accumulation of Ca^2+^ took place in the cleft space with a very similar time course as the former depletion. This compares well with Ca^2+^ depletion measured in the perfused right ventricular tissue of rabbits (amounting to 0.3 to 0.5 mM) reported by Hilgemann and Langer [22] in a train of eight stimulations at 0.5-s intervals applied from rest.

## 3. Discussion

This study demonstrates that ion concentration changes in the t-system of human cardiomyocytes, though small compared to those observed in skeletal muscle [23,24], are not negligible and should be considered in a detailed description of a human ventricular myocyte as a factor contributing to the control of Ca^2+^ turnover and cardiac contraction. This confirms our previous results from simulations in models of animal cardiac ventricular myocytes of guinea-pig [14] and rat [16]. In addition, [Ca^2+^] changes occurring in the limited space of t-tubules are potentiated by [Ca^2+^] changes in the intercellular clefts, primarily during transients caused by changes in stimulation frequency.

### 3.1. Validation of the Model

**Figure 8.**
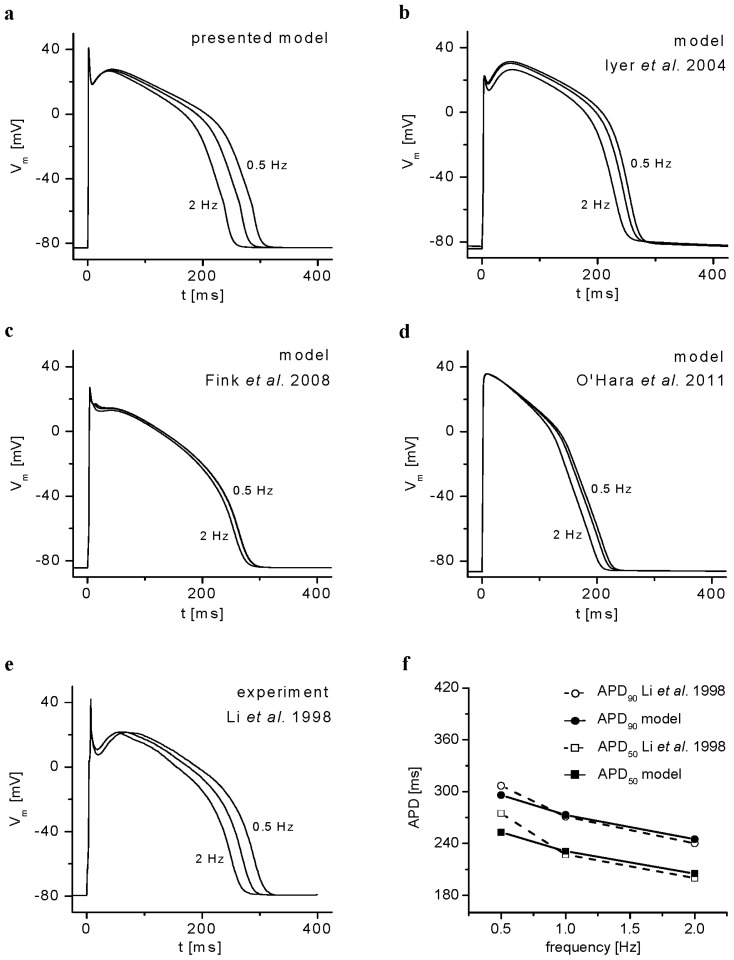
Comparison of model reconstructions of steady-state AP waveforms recorded at 0.5, 1 and 2 Hz stimulation in a human subepicardial cell by Li *et al.* [25]. The following models were used: (**a**) the present model; (**b**) the model of Iyer *et al.* [5]; (**c**) the model of Fink *et al.* [6] and (**d**) the model of O’Hara *et al.* [8]; (**e**) Representative AP waveforms digitized from Li *et al.* [25]; and (**f**) Comparison of values of APD_50_ and APD_90_ (action potential duration at 50% and 90% repolarization, respectively) at 0.5, 1 and 2 Hz obtained from the experimental data of Li *et al.* [25] and the present model. In the models, 15 APs were elicited at each stimulation rate, as done in [25].

The ability of the present model to simulate changes of APs recorded in experiments in human cardiomyocytes at different stimulation frequencies [25] is demonstrated in Figure 8. This frequency dependence of APD was shown to be associated primarily with modulation of *I*_CaL_[26]. For the sake of comparison, we reconstructed the frequency dependence of AP configuration also by using three other models of human ventricular myocytes recently published by Iyer *et al.* [5], Fink *et al.* [6] and O’Hara *et al.* [8]. To strictly meet the experimental conditions specified in [25], we applied only 15 stimulation pulses, and [Na^+^]_i_ and [K^+^]_i_ were fixed at 10 and 130 mM, respectively (ion concentrations used in the pipette solution). Surprisingly, the frequency-dependent changes of AP duration in the models ([6] and [8]) were quite small. Larger changes in AP duration could be observed in these two models only when a substantially higher number of stimulation pulses were applied (e.g., 300 pulses) and when the simulations were done without fixation of [Na^+^]_i_ and [K^+^]_i_.

**Figure 9.**
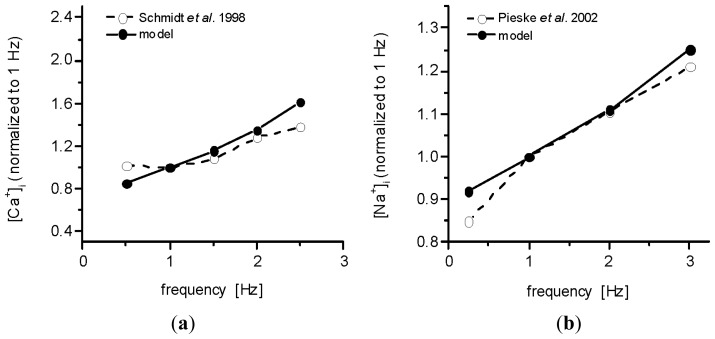
Comparison of normalized data showing the frequency-dependent increase of intracellular diastolic ion concentrations in the present model and in the experimental studies. (**a**) [Ca^2+^]_i_ (100 APs elicited at each stimulation rate); data published by Schmidt *et al.* [27]; and (**b**) [Na^2+^]_i_ (600 APs elicited at each stimulation rate); data published by Pieske *et al.* [28].

The ability of our model to simulate the frequency-dependent changes of intracellular ion concentrations measured in human preparations by Schmidt *et al.* [27] ([Ca^2+^]_i_) and Pieske *et al.* [28] ([Na^+^]_i_) is illustrated in Figure 9. The data are normalized to the values at 1 Hz. The resting value of [Ca^2+^]_i_ in our model (63.6 nM) agrees well with the experimental data of Beuckelmann *et al.* [29], and that of [Na^+^]_i_ (9.3 mM) is in the range of the experimentally observed values of Pieske *et al.* [30].

### 3.2. Conditions of Model Stability

The model showed long-term stability, as demonstrated in Figure 11. For all types of ions, the conditions of stability required that, at steady state, the net amount of substance transferred across the cell membrane during one period (*T*) at any frequency equaled zero. Particularly for calcium ions, this condition can be expressed as:

(1) $$n_{\text{Ca},\text{net},\text{s}}+n_{\text{Ca},\text{net},\text{t}}=0$$

where *n*_Ca,net,s_ and *n*_Ca,net,t_ denote net amounts of Ca^2+^ ions transferred during each stimulation period, *T*, across the surface and the t-tubular membrane, respectively. They can be expressed as the time integrals of fluxes *J*_Ca,net,s_ and *J*_Ca,net,t_ over the stimulation period (*T*) or as a sum of integrals of all ionic currents taking a share in transmembrane Ca^2+^ transport through each membrane pool.

(2) $$\begin{array}{l} n_{\text{Ca},\text{net},\text{t}}=\int_{T}J_{\text{Ca},\text{net},\text{t}}=\frac{1}{2F}\int_{T}\left(I_{\text{CaL},\text{t}}-2I_{\text{NaCa},\text{t}}+I_{\text{Cab},\text{t}}+I_{\text{pCa},\text{t}}\right)\;dt\\ n_{\text{Ca},\text{net},\text{s}}=\int_{T}J_{\text{Ca},\text{net},\text{s}}=\frac{1}{2F}\int_{T}\left(I_{\text{CaL},\text{s}}-2I_{\text{NaCa},\text{s}}+I_{\text{Cab},\text{s}}+I_{\text{pCa},\text{s}}\right)\;dt \end{array}$$

All currents transporting Ca^2+^ across both membrane systems are modulated primarily by changes in [Ca^2+^]_i_, but, to a certain degree, also by changes in [Ca^2+^]_t_ and [Ca^2+^]_c_. Those modulations represent a complex feedback system maintaining SR Ca^2+^ load and, hence, the magnitude of the [Ca^2+^]_i_-transient, at a level that guarantees steady-state Condition (1) at any frequency. During a transient (evoked, e.g., by a stepwise change in stimulation frequency), Condition (1) is disturbed, until a new steady state is attained.

### 3.3. The Effects of Extracellular Ion Concentration Changes Under Steady-State and Non-Steady-State Conditions

Under steady state at any frequency, the mean ion concentrations in the intercellular clefts are equilibrated with concentrations in the bulk. Not surprisingly, inclusion of the clefts into the model only minutely affected the simulated results of intracellular Ca^2+^ concentration changes ([Ca^2+^]_i_ and [Ca^2+^]_NSR_) at steady state (Figure 3). When exploring steady-state effects in the model, ionic currents and the quantities describing Ca^2+^ turnover remained practically unaltered when ionic concentrations in the intercellular clefts were fixed at bulk concentrations (not illustrated). In contrast, in the t-tubules, the mean Ca^2+^ concentration differs from the bulk concentration as a consequence of the sustained flux, *J*_Ca,net,t_ (cycling between the tubular and surface membrane [16]). In addition, activity-induced steady-state oscillations in the clefts are relatively small as compared to those in the t-tubular lumen (Figure 2), due to the larger volume of the intercellular cleft and the smaller Ca^2+^ channel density at the surface membrane. Thus, the currents mediating transmembrane Ca^2+^ movements in the course of each cycle at steady state are more affected by a depletion of extracellular Ca^2+^ (which reaches a maximum within one cycle during activation of *I*_CaL_) at the t-tubular membrane than at the surface membrane.

Although the fractions of the main ion transporting proteins localized in the tubular membrane have been quantified in some experimental animals (for reviews, see [31,32]), similar data related to human cardiomyocytes are still missing. Therefore, we investigated a possible influence of varying the fractions of Ca^2+^ transporters in the t-tubular membrane on the modulation of intracellular Ca^2+^-transient (represented by Δ[Ca^2+^]_i,area,rel_). The graphs in Figure 4 demonstrate the steady-state effect for two values of *f*_CaL,t_ assessed in guinea-pig (0.64) and rat (0.8) cardiomyocytes. As *I*_CaL,t_ causes Ca^2+^ depletion in the t-tubules, it is not surprising that a higher *f*_CaL,t_ corresponds to a lower amount of Ca^2+^ in the intracellular compartments reflected by Δ[Ca^2+^]_i,area,rel_. Conversely, raising fractions *f*_NaCa,t_ and *f*_pCa,t_ attenuates changes in Ca_i_-transient, because *I*_NaCa,t_ and *I*_pCa,t_ tend to enhance the mean value of [Ca^2+^]_t_. The simulations suggest that the changes in intracellular Ca^2+^-transient may be significant if, simultaneously, *f*_CaL,t_ was high (≥0.6) and *f*_pCa,t_ low (≤0.2).

In contrast, under non-steady-state conditions, when sudden changes in heart rate were simulated, the intercellular cleft space significantly affected Ca^2+^ handling and APs (Figures 5 and 6). This was caused by a significant transient depletion of extracellular Ca^2+^ concentrations in the cleft space and, consequently, in t-tubules. This process resulted from the initial fast increase of Ca^2+^ input into the cell (within several stimuli), since, during each shortened stimulation period after transition to 2.5 Hz, Ca^2+^ input via *I*_CaL_ transiently exceeded Ca^2+^ output via *I*_NaCa_ and *I*_pCa_. Restoration of equilibrium between the clefts and the bulk space required tens of seconds.

Transient accumulation of extracellular K^+^ in response to sudden changes of stimulation frequency (Figure 7) was evidenced as parallel changes in resting membrane voltage in agreement with experimental results [21]. It also played a role in the reduction of Ca^2+^-transient (not shown), but its effect was substantially smaller than the effect of Ca^2+^ depletions. The effects of minute relative variations of [Na^+^]_i_ were negligible.

### 3.4. Cardiomyopathies Alter the Function and Structure of Tubular System

As documented by numerous studies, cardiac diseases, like ischemic or dilated heart failure, affect the structure and function of the t-tubular system, both in animal [33–35] and human cardiomyocytes [36–38] (for a review, see [39]). The myocardial remodeling includes a reduced number of t-tubule openings [38,40], altered geometry (diameter and length) [36,41] and redistribution of ion transporting proteins between the surface and tubular membrane, namely L-type Ca-channels and Na/Ca-exchange proteins [42]. As a consequence, alteration in Ca^2+^ turnover and EC coupling can be expected. Intercellular clefts can also be modified by, e.g., hypertrophic cardiomyopathy [43].

The purpose of the present model study was to estimate the impact of ionic changes in extracellular spaces on APs, ionic currents and intracellular Ca^2+^ handling in normal human cardiomyocytes at different frequencies. Our aim in a future work will be to utilize the present model to investigate the consequences of the above-mentioned changes of ion concentrations in extracellular spaces occurring under pathological conditions.

### 3.5. Limitations of the Study

#### 3.5.1. Inhomogeneities Along the T-Tubules

The distribution of ion channels along the t-tubules is considered uniform in our model. However, the tubular system comprises both transverse and longitudinal extensions, and ion channels are probably unevenly distributed among these two membrane pools [17]. Further, channels are likely clustered in specialized regions [44]. The concentration of Ca^2+^ channels, at the limited region of the t-tubules that contribute to the dyads, would result in larger local depletions in luminal Ca^2+^, as modeled using partial differential equations [45].

#### 3.5.2. Deformation of the T-Tubules

Deformation of the t-tubules during activity [46] is likely to modulate the magnitude of ionic currents through mechano-sensitivity. In addition, the t-tubules’ diameter is narrowed under high blood pressure or under contracture [47]. Such a change in volume during a cardiac cycle would cause fluid exchange between the t-tubule lumen and the interstitial space and help in damping concentration changes. The magnitude of this effect still remains to be evaluated for human ventricular myocytes.

#### 3.5.3. Cellular Subtypes and Regional Variations

Subtypes of ventricular cardiac myocytes (epicardial, mid-myocardial or endocardial) with different ion channel densities were not considered in our study, as their description in human cardiac ventricular myocytes is not fully documented. It was also shown that the amount of t-tubules-SR junctions (but not the volume fraction of t-tubules) varied among different regions of rabbit ventricular myocardium that displayed a difference in AP configuration [48,49]. For these reasons, this study did not aim to provide a precise quantitative view of the behavior of human cardiac cells, but rather, an average representation that will be refined when a more detailed description of human ventricular cells and tissue becomes available.

## 4. Methods

**Figure 10.**
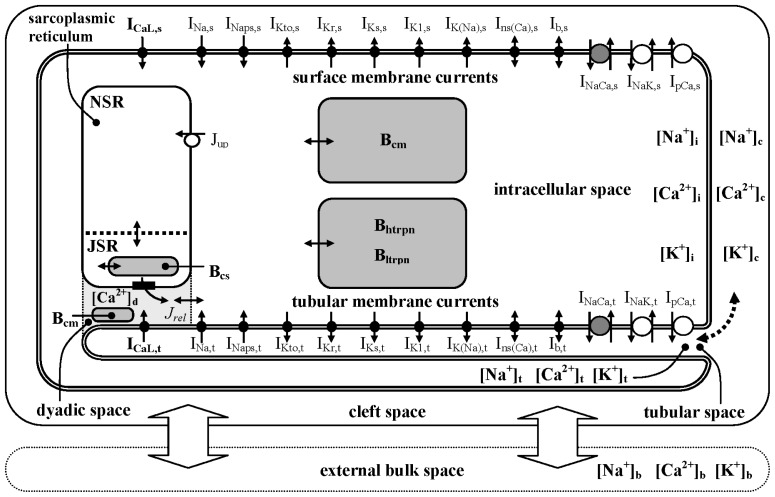
Schematic diagram of the human ventricular cell model used in the present study. The description of the electrical activity of surface (s) and t-tubular (t) membranes comprises formulations of the following ion currents: fast sodium current (*I*_Na_), persistent sodium current (*I*_Naps_), L-type calcium current (*I*_CaL_), transient outward potassium current (*I*_Kto_), rapid and slow components of delayed rectifier potassium current (*I*_Kr_ and *I*_Ks_), inward rectifying potassium current (*I*_K1_), background currents (*I*_b_), sodium-activated potassium current (*I*_K(Na)_), calcium-activated non-specific current (*I*_ns(Ca)_), sodium-calcium exchange current (*I*_NaCa_), sodium-potassium pump current (*I*_NaK_) and calcium pump current (*I*_pCa_). The intracellular space contains the cytosol (i), the dyadic space (d), network and junctional compartments of sarcoplasmic reticulum (NSR, JSR) and endogenous Ca^2+^ buffers [calmodulin (B_cm_), troponin (B_htrpn_, B_ltrpn_) and calsequestrin (B_cs_)]. The t-tubular space is partitioned into nine concentric cylindrical segments (details are given in the Supplementary Information). *J*_up_ represents Ca^2+^ flow via SR Ca^2+^ pump and the small filled rectangles in JSR membrane ryanodine receptors. The small bi-directional arrows denote Ca^2+^ diffusion. Ion diffusion between the t-tubular and cleft spaces is represented by the dashed arrow and between cleft and external bulk spaces by thick arrows.

The structure of the model (Figure 10) is similar to that of our recent models incorporating t-tubules. From the two existing species-specific models including t-tubular spaces (rat [16] and guinea pig [14]), the properties of guinea pig cardiomyocytes are nearer to the human one. Therefore, we started with our guinea pig model that we then modified, with respect to the recently published morphological data [5,15], the known properties of ion transfer mechanisms [4,5], the configuration of action potentials (APs) [25] and the changes of intracellular ion concentrations in human ventricular cardiomyocytes [27,28].

The principal modifications include: (i) replacement of the description of *I*_Na_, *I*_CaL_ and *I*_K1_ by the formulations used by Iyer *et al.* [5] and those of *I*_Kr_, *I*_Ks_ and *I*_Kto_ by the simpler formulations of Ten Tusscher *et al.* [4]; (ii) reformulation of intracellular Ca^2+^ handling mainly on the basis of its description in [5]; (iii) partitioning of t-tubular space along its radius into nine concentric cylindrical segments [16] and readjustment of geometrical parameters of t-tubules to meet the recent experimental data from human hearts [15]; and (iv) formulation of a single-layer cleft space and ion exchange with an external bulk space on the basis of the data in [2].

Because of the paucity of experimental data from human ventricular myocytes, the time constants describing ion exchange between t-tubular and cleft spaces and also t-tubular fractions of ion transporters related to *I*_Na_, *I*_CaL_, *I*_K1_ and *I*_pCa_ were preliminarily set to be consistent with data obtained from guinea-pig [14]. The t-tubular fractions of other transporters were set to 0.56 (the fraction of membrane area in t-tubules) on the assumption of their uniform density over the whole cell membrane. The model using these settings is referred to as the “basic model” in the text. Some parameters, so far undefined for human ventricular myocytes, such as tubular fractions of L type Ca^2+^-channels (*f*_CaL,t_), Na^+^-Ca^2+^ exchangers (*f*_NaCa,t_) and the sarcolemmal SR Ca^2+^ pump (*f*_pCa,t_), were, however, considered as variable in some simulations to evaluate their importance. All details related to the development of the model and its full mathematical description are given in the Supplementary Information (model parameters are summarized in Figure S1 and Tables S2–S10).

**Figure 11.**
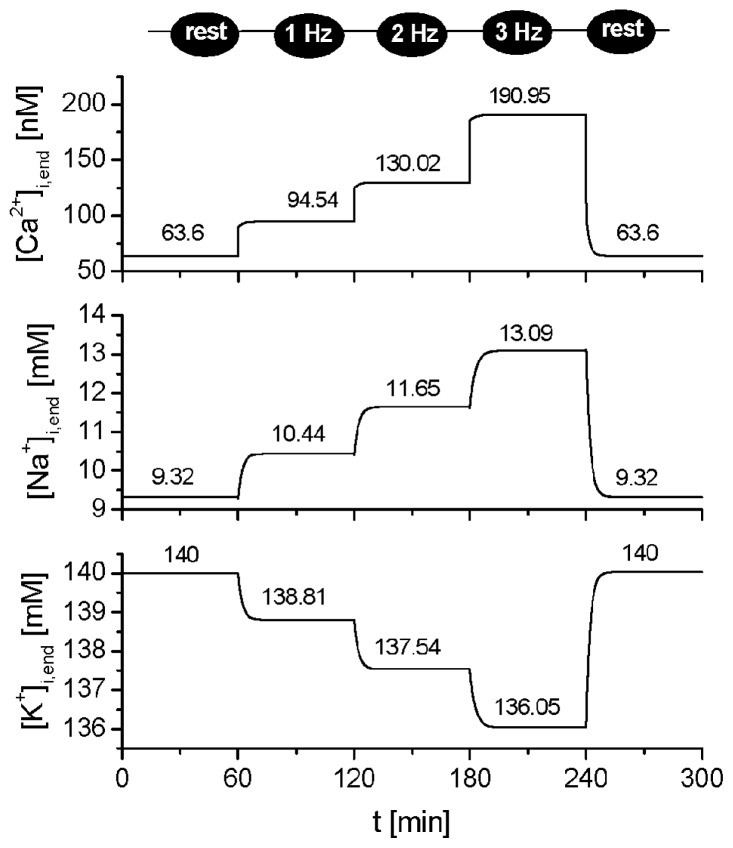
The stability of the model showing cytoplasmic end-diastolic [Ca^2+^] (**top**), [Na^+^] (**middle**) and [K^+^] (**bottom**) during a 5-h cell lifetime run during which the model cell was initially at rest and then stimulated at 1, 2 and 3 Hz and returned to rest for one hour, as indicated on the top of the panel.

The model was implemented in MATLAB 7.2 (MathWorks, Natick, MA, USA), and the numerical computation of the system of 98 nonlinear differential equations was performed using the solver for stiff system ODE-15s. The stability of the model with time is demonstrated in Figure 11. To achieve steady states in Figures 1–4, the model was run for 1200 s at each stimulation rate.

To compare the model results with the behavior of other models of human ventricular cells published so far [5,6,8], we used the computational environment for cellular modelling, COR v. 0.9.31.1409 (Dr. Alan Garny), and the available CellML codes of the models.

## 5. Conclusions

The available data related to human cardiac ventricular cells were used to design the first mathematical model of human ventricular cardiomyocyte incorporating t-tubular and intercellular cleft spaces. The model was used to study the physiological consequences of activity-related ion concentration changes in these extracellular spaces. Some of them (particularly, the changes of the intracellular Ca^2+^-transient) were explored as a function of a few crucial parameters, still not quantified in human cardiac cells. In the present state, the model visualizes the activity-related accumulation-depletion of Ca^2+^ and K^+^ ions in t-tubular and interstitial extracellular spaces. The results suggest that cumulative calcium depletion in the limited extracellular spaces can affect transmembrane Ca^2+^ currents, the duration of AP and the cytosolic calcium-transients that govern cellular contraction. Further experimental work is needed to evaluate the missing values describing the distribution of ion transporters in the cell membrane and the rate of ion exchanges between the extracellular compartments of human cardiomyocytes.

## Supplementary Information



## Appendix

**Table:**

Symbol	Definition
Abbreviations used in the text	
AP	Action potential
APD	Action potential duration
EC	Excitation-contraction
SR	Sarcoplasmic reticulum
NSR	Network compartment of SR
JSR	Junctional compartment of SR
Variables used in the text	
*V*_m_	Membrane voltage
*I*_Na_	Fast Na^+^ current
*I*_CaL_	L-type Ca^2+^ current
*I*_Naps_	Persistent Na^+^ current
*I*_Kto_	Transient outward K^+^ current
*I*_Kr_	Rapid delayed rectifier K^+^ current
*I*_Ks_	Slow delayed rectifier K^+^ current
*I*_K1_	Inward rectifying K^+^ current
*I*_K(Na)_	Sodium-activated K^+^ current
*I*_ns(Ca)_	Calcium-activated non-specific current
*I*_b_	Background current
*I*_NaCa_	Na^+^-Ca^2+^ exchange current
*I*_NaK_	Na^+^-K^+^ pump current
*I*_pCa_	Ca^2+^ pump current
*I*_x,s_	Surface membrane component of current *x*
*I*_x,t_	Tubular membrane component of current *x*
*J*_up_	Ca*^2+^* flow via SR Ca^2+^ pump
*J*_x,net,t_	Net flux of ion *x* through t-tubular membrane
*J*_x,net,s_	Net flux of ion *x* through surface membrane
*n*_x,net,t_	Net amount of ion *x* transferred via t-tubular membrane during a cycle
*n*_x,net,s_	Net amount of ion *x* transferred via surface membrane during a cycle
[*x*]_b_	Free concentration of substance *x* in external bulk space
[*x*]_c_	Free concentration of substance *x* in cleft space
[*x*]_t_	Free concentration of substance *x* in t-tubular space
[*x*]_d_	Free concentration of substance *x* in dyadic space
[*x*]_i_	Free concentration of substance *x* in cytosol
[*x*]_end_	Free concentration of substance *x* at the end of cycle
[*x*]_mean_	Mean free concentration of substance x during a cycle
[*x*]_NSR_	Free concentration of substance *x* in NSR
[*x*]_JSR_	Free concentration of substance *x* in JSR
Parameters/constants used in the text	
*f*_i,t_	Fraction of ion transporters *i* in t-tubular membrane
τ_x,ct_	Time constant of exchange of ion *x* between intercellular clefts and t-tubules
