# Supplementary material for: Effect of Ion Concentration Changes in the Limited Extracellular Spaces on Sarcolemmal Ion Transport and Ca2+ Turnover in a Model of Human Ventricular Cardiomyocyte
Source: Int J Mol Sci. 2013 Dec 13;14(12):24271–92. doi: 10.3390/ijms141224271 (PMC3876110; doi:10.3390/ijms141224271)
Supplement: Supplementary file 1 [file ijms-14-24271-s001.pdf]

Supplementary Information

Description of Model Design

Geometrical Parameters of Cellular Compartments

The total volume of the model cell (V_{cell}) was set to 20.9 pL. The fractional volumes of intracellular compartments (myoplasm 0.68, NSR 0.055, JSR 0.0042 and dyadic space $f_{Ca,t}$ × 0.000032) were adjusted to be consistent with the model of human ventricular epicardial myocyte published by Iyer *et al.* [5]. The total membrane area of 15,000 μm^2 corresponds with the membrane capacitance of 150 pF. The dyadic space is described only at the t-tubules because of their major role in excitation-contraction coupling [50].

The total volume of the t-tubular system in the model cell represents the sum of the volumes of all t-tubules (their density in surface membrane was set to 0.19 per μm^2 [38]). To keep all geometric parameters of the model cell consistent with surface/volume ratios (A_{tot}/V_{cell} = 0.72 μm^2/μm^3, A_{tub}/V_{cell} = 0.403 μm^2/μm^3, A_{surf}/V_{cell}= 0.327 μm^2/μm^3), mean radius of the t-tubules (210 nm) and fractional area of t-tubular membrane (0.56) published by Ohler *et al.* [15], the fractional volume of t-tubular system must have been reduced from 0.0867 to 0.042 [15]. To simulate the ion gradient in t-tubular lumen, the system ("each t-tubule") was partitioned into nine concentric cylindrical segments St_1, St_2,…, St_9 (Figure S1). To increase the accuracy of computation of ion concentration changes close to the t-tubular membrane, the volumes of segments St_1−St_8 were set to 3% of the total tubular volume; further reduction did not significantly alter the results. The outer radius of each segment is listed in the table of Figure S1.

Figure S1. Schematic representation of part of the model t-tubule. The tubular lumen is partitioned into nine concentric cylindrical segments St_1, St_2,…, St_9. Segments St_1–St_8 (shown in grey) occupy 25.3 times smaller volume than the segment St_9 (white centre). The table shows the outer radii of individual tubular segments and their fractional volumes.

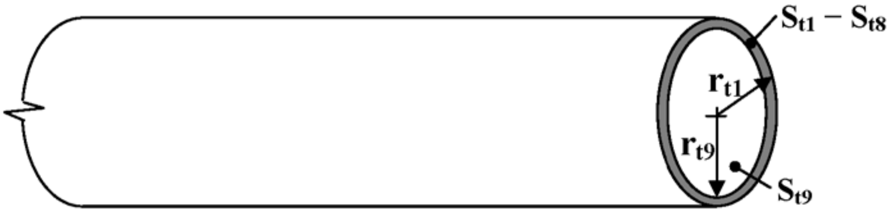

Segment	Radius	Fract. volume
S_{t1}	210.0 nm	0.03
S_{t2}	206.8 nm	0.03
S_{t3}	203.6 nm	0.03
S_{t4}	200.3 nm	0.03
S_{t5}	197.0 nm	0.03
S_{t6}	193.6 nm	0.03
S_{t7}	190.2 nm	0.03
S_{t8}	186.7 nm	0.03
S_{t9}	183.1 nm	0.76

Membrane Currents

The description of principal membrane currents was modified to be consistent with recently published models of human cardiomyocytes. The formulations of I_{Na}, I_{Ca} and I_{K1} were adopted from Iyer *et al.* [5]. To describe I_{Kr}, I_{Ks} and I_{Kto} the simplified formulations published by Ten Tusscher *et al.* [4] were used. Other currents such as I_{NaCa}, I_{NaK}, I_{pCa}, I_{Naps}, $I_{K(Na)}$, $I_{ns(Ca)}$ and I_b remained unchanged [14] and I_{Kp} and $I_{K(ATP)}$ were omitted.

The maximum conductivities, current densities or permeabilities of ion transport systems (Table S1) were adopted from the above cited papers and, if needed, adjusted for the model to generate physiological configuration of APs as well as physiological levels of intracellular ion concentrations at rest and during activity [27–30]. An acceptable fit of the model simulations with experimental data of Li *et al.* [25] required also a slight reformulation of the steady-state voltage dependent inactivation of I_{Ca} (y_∞) as:

$$y_\infty = \frac{0.82}{1 + e^{(V_m+28.5)/7.8}} + 0.1 \tag{1}$$

and of the time constant of I_{to}-inactivation (τ_s) as:

$$\tau_s = 0.085\,\mathrm{e}^{-(V_m+45)^2/320} + \frac{0.005}{1+\mathrm{e}^{(V_m-63)/5}} + 0.003 \tag{2}$$

Table S1. Maximum conductivity ($g_{X,max}$), current density ($I_{X,max}$) or permeability (P_x) of ion transport systems used in the model. The values are related to the total membrane area. $f_{X,t}$ represents the t-tubule fraction of the ion transporter underlying current I_X.

$g_{Na,max}$	25 mS cm^{-2}	$f_{Na,t}$	0.57
$g_{Naps,max}$	0.01 mS cm^{-2}	$f_{Naps,t}$	0.56
P_{CaL}	0.00185 cm s^{-1}	$f_{CaL,t}$	0.64
P_{KL}	0.0000032 cm s^{-1}	$f_{KL,t}$	0.64
$g_{Kto,max}$	0.132 mS cm^{-2}	$f_{Kto,t}$	0.56
$g_{Kr,max}$	0.038 mS cm^{-2}	$f_{Kr,t}$	0.56
$g_{Ks,max}$	0.040 mS cm^{-2}	$f_{Ks,t}$	0.56
$g_{K1,max}$	0.125 mS cm^{-2}	$f_{K1,t}$	0.80
$g_{K(Na),max}$	0.129 mS cm^{-2}	$f_{K(Na),t}$	0.56
$g_{Nab,max}$	0.141 μS cm^{-2}	$f_{Nab,t}$	0.56
$g_{Cab,max}$	2.413 μS cm^{-2}	$f_{Cab,t}$	0.56
$P_{ns(Ca)}$	1.75 nm s^{-1}	$f_{ns(Ca),t}$	0.56
$k_{NaCa,max}$	0.15 nA cm^{-2} mM^{-4}	$f_{NaCa,t}$	0.56
$I_{NaK,max}$	0.975 μA cm^{-2}	$f_{NaK,t}$	0.56
$I_{pCa,max}$	1.725 μA cm^{-2}	$f_{pCa,t}$	0.20

Because of the paucity of experimental data related to distribution of ion transporters between the surface and t-tubular membrane in human ventricular cells the t-tubular fractions of ion transporters related to currents I_{Na}, I_{Ca}, I_{K1} and I_{pCa} (Table S1) were preliminarily set to be consistent with guinea pig model [14]. T-tubular fractions of other transporters were set to 0.56 assuming their uniform distribution over the whole membrane.

Intracellular Ca^{2+}- Handling

The formulation of intracellular Ca^{2+} handling is based on the description used by Iyer *et al.* [5]. However, to prevent the intracellular Ca^{2+} overload and irregular Ca^{2+} release from JSR at higher stimulation rates, a readjustment of some parameters/constants was needed. It included: (i) increase of forward and reverse rate parameters of SR Ca^{2+} pump (v_{maxf} and v_{maxr}) to 0.25 mM s^{-1} and 0.75 mM s^{-1} respectively; (ii) decrease of on- and off-rate constants controlling adaptation of RyR channels (K_c^+ and K_c^-) to 0.1 ms^{-1} and 0.0008 ms^{-1} respectively; (iii) increase of the time constant of Ca^{2+} transfer from NSR to JSR (τ_{tr}) to 20 ms; (iv) decrease of the time constant of Ca^{2+} transfer from the dyadic space into the cytosol (τ_{xfer}) to 18 ms.

Ion Exchange Between t-Tubular Segments and with the Extracellular Cleft Space

No data are currently available about the rate of ion diffusion within tubular lumen and between t-tubular and extracellular spaces. For this reason, the time constants controlling the ion exchange between cleft and t-tubular spaces are preliminarily adjusted to the same values as in guinea pig model, *i.e.*: $\tau_{Ca,ct}$ = 240 ms, $\tau_{K,ct} = \tau_{Na,ct}$ = 200 ms [14].

The time constants controlling Ca^{2+} and K^+ exchange between the individual tubular segments were computed by multiplying the $\tau_{Ca,ct}$ and $\tau_{K,ct}$ by a factor taking into account the different diffusion area between individual segments and, in segments S_{t8} and S_{t9}, their different volumes. The resulting formulation is:

$$\tau_{X,St,n+1} = \tau_{X,extt} \cdot \frac{A_{base,n}}{A_{boundary,n}} \cdot \frac{V_{St,n+1}}{V_{St,n}} \quad (3)$$

where $\tau_{X,St,n+1}$ represents the time constant controlling the rate of diffusion of ion X from segment n to segment *n*+1 and the parameters $A_{\text{base},n}$, $A_{\text{boundary},n}$, $V_{\text{St},n+1}$ and $V_{\text{St},n}$ represent the area of segment *n* in the tubular mouth, area of the boundary between segments *n* and *n*+1, volume of segment *n*+1 and volume of segment *n*, respectively.

Because the concentration changes of tubular Na^+ are minimal, they are described in a single t-tubule compartment as in our guinea pig model [14].

Extracellular Cleft Space

Extracellular cleft space is described as a single compartment surrounding the model cell. The volume of this compartment (1.932 pl, 13.6% of total cytosolic volume) as well as the time constants controlling the rate of ion exchange between this compartment and bulk space ($\tau_{Ca,bc}$ = 24.7 s, $\tau_{Na,bc}$ = 14.3 s and $\tau_{K,bc}$ = 10 s) were set to be consistent with the model of human atrial myocyte published by Nygren *et al.* [2]. The bulk concentrations of Ca^{2+}, Na^+ and K^+ were set to 2 mM, 140 mM and 5.4 mM, respectively.

Model Equations and Parameters

The basic units in which the equations were solved are: mV for membrane voltage, μA for membrane currents, mS for conductivities, ml s^{-1} for permeabilities, μmol s^{-1} for ionic fluxes, mmol L^{-1} for ionic concentrations, ml for volumes and s for time. The suffix "x" stands for either surface ("s") or t-tubular ("t") membrane variables, and the suffix "o" correspondingly stands for ion concentrations either in intercellular cleft space ("c") or in the first t-tubular segment ("t_1"). The physical constants and geometrical parameters of the model cell are specified in Tables S2 and S3, respectively.

Membrane Currents

The values of parameters and constants used in the description of membrane currents are listed in Tables S4 and S5.

Na^+ Current, I_{Na}

$$I_{\mathrm{Na,x}} = g_{\mathrm{Na,x}}\left(O_{\mathrm{1Na,x}} + O_{\mathrm{2Na,x}}\right)(V_{\mathrm{m,x}} - E_{\mathrm{Na,x}}) \tag{4}$$

$$\frac{\mathrm{d}C_{\mathrm{0Na,x}}}{\mathrm{d}t} = -\left(4\alpha_{\mathrm{x}} + c_{\mathrm{n}}\right)C_{\mathrm{0Na,x}} + \beta_{\mathrm{x}}C_{\mathrm{1Na,x}} + c_{\mathrm{f}}CI_{\mathrm{0Na,x}} \tag{5}$$

$$\frac{\mathrm{d}C_{\mathrm{1Na,x}}}{\mathrm{d}t} = -\left(\beta_{x} + c_{\mathrm{n}}\cdot a + 3\alpha_{\mathrm{x}}\right)C_{\mathrm{1Na,x}} + 4\alpha_{\mathrm{x}}C_{\mathrm{0Na,x}} + 2\beta_{\mathrm{x}}C_{\mathrm{2Na,x}} + \left(c_{\mathrm{f}}/a\right)CI_{\mathrm{1Na,x}} \tag{6}$$

$$\frac{\mathrm{d}C_{\mathrm{2Na,x}}}{\mathrm{d}t} = -\left(2\beta_{\mathrm{x}} + c_{\mathrm{n}}\cdot a^{2} + 2\alpha_{\mathrm{x}}\right)C_{\mathrm{2Na,x}} + 3\alpha_{\mathrm{x}}C_{\mathrm{1Na,x}} + 3\beta_{x}C_{\mathrm{3Na,x}} + \left(c_{\mathrm{f}}/a^{2}\right)CI_{\mathrm{2Na,x}} \tag{7}$$

$$\frac{\mathrm{d}C_{\mathrm{3Na,x}}}{\mathrm{d}t} = -\left(3\beta_{\mathrm{x}} + c_{\mathrm{n}}\cdot a^{3} + \alpha_{\mathrm{x}}\right)C_{\mathrm{3Na,x}} + 2\alpha_{\mathrm{x}}C_{\mathrm{2Na,x}} + 4\beta_{\mathrm{x}}C_{\mathrm{4Na,x}} + \left(c_{\mathrm{f}}/a^{3}\right)CI_{\mathrm{3Na,x}} \tag{8}$$

$$\frac{\mathrm{d}C_{\mathrm{4Na,x}}}{\mathrm{d}t} = -\left(4\beta_{\mathrm{x}} + c_{\mathrm{n}}\cdot a^{4} + \gamma_{\mathrm{x}} + \eta_{\mathrm{x}}\right)C_{\mathrm{4Na,x}} + \alpha_{\mathrm{x}}C_{\mathrm{3Na,x}} + \delta_{\mathrm{x}}O_{\mathrm{1Na,x}} + \nu_{\mathrm{x}}O_{\mathrm{2Na,x}} + \left(c_{\mathrm{f}}/a^{4}\right)CI_{\mathrm{4Na,x}} \tag{9}$$

$$\frac{\mathrm{d}O_{\mathrm{1Na,x}}}{\mathrm{d}t} = -\left(\delta_{\mathrm{x}} + \varepsilon + o_{\mathrm{n,x}}\right)O_{\mathrm{1Na,x}} + \gamma_{\mathrm{x}}C_{\mathrm{4Na,x}} + \omega_{\mathrm{x}}O_{\mathrm{2Na,x}} + o_{\mathrm{f,x}}OI_{\mathrm{Na,x}} \tag{10}$$

$$\frac{\mathrm{d}O_{\mathrm{2Na,x}}}{\mathrm{d}t} = -\left(\omega_{\mathrm{x}} + \nu_{\mathrm{x}}\right)O_{\mathrm{2Na,x}} + \varepsilon O_{\mathrm{1Na,x}} + \eta_{\mathrm{x}}C_{\mathrm{4Na,x}} \tag{11}$$

$$\frac{\mathrm{d}CI_{\mathrm{0Na,x}}}{\mathrm{d}t} = -\left(c_{\mathrm{f}} + 4\alpha_{\mathrm{x}}a\right)CI_{\mathrm{0Na,x}} + \left(\beta_{\mathrm{x}}/a\right)CI_{\mathrm{1Na,x}} + c_{\mathrm{n}}C_{\mathrm{0Na,x}} \tag{12}$$

$$\frac{\mathrm{d}CI_{\mathrm{1Na,x}}}{\mathrm{d}t} = -\left(\beta_{\mathrm{x}}/a + 3\alpha_{\mathrm{x}}a + c_{\mathrm{f}}/a\right)CI_{\mathrm{1Na,x}} + 4\alpha_{\mathrm{x}}aCI_{\mathrm{0Na,x}} + \left(2\beta_{\mathrm{x}}/a\right)CI_{\mathrm{2Na,x}} + c_{\mathrm{n}}aC_{\mathrm{1Na,x}} \tag{13}$$

$$\frac{\mathrm{d}CI_{\mathrm{2Na,x}}}{\mathrm{d}t} = -\left(2\beta_{\mathrm{x}}/a + 2\alpha_{\mathrm{x}}a + c_{\mathrm{f}}/a^{2}\right)CI_{\mathrm{2Na,x}} + 3\alpha_{\mathrm{x}}aCI_{\mathrm{1Na,x}} + \left(3\beta_{\mathrm{x}}/a\right)CI_{\mathrm{3Na,x}} + c_{\mathrm{n}}a^{2}C_{\mathrm{2Na,x}} \tag{14}$$

$$\frac{\mathrm{d}CI_{\mathrm{3Na,x}}}{\mathrm{d}t} = -\left(3\beta_{\mathrm{x}}/a + \alpha_{\mathrm{x}}a + c_{\mathrm{f}}/a^{3}\right)CI_{\mathrm{3Na,x}} + 2\alpha_{\mathrm{x}}aCI_{\mathrm{2Na,x}} + \left(4\beta_{\mathrm{x}}/a\right)CI_{\mathrm{4Na,x}} + c_{\mathrm{n}}a^{3}C_{\mathrm{3Na,x}} \tag{15}$$

$$\frac{dCI_{4Na,x}}{dt} = -\left(4\beta_x / a + \gamma\gamma_x + c_f / a^4\right) CI_{4Na,x} + \alpha_x a CI_{3Na,x} + \delta\delta_x OI_{Na,x} + c_n a^4 C_{4Na,x} \tag{16}$$

$$\frac{dOI_{Na,x}}{dt} = -\left(\delta\delta_x + o_{f,x}\right) OI_{Na,x} + \gamma\gamma_x CI_{4Na,x} + o_{n,x} O_{1Na,x} \tag{17}$$

$$E_{Na,x} = \frac{RT}{F} ln\left(\frac{\left[Na^+\right]_o}{\left[Na^+\right]_i}\right) \tag{18}$$

$$\alpha_x = 2.0842 \cdot 10^{10} T e^{\left(-7907.5+6.013T+3.3205V_{m,x}\right)/T} \tag{19}$$

$$\beta_x = 2.0842 \cdot 10^{10} T e^{\left(-32768+83.19T-28.985V_{m,x}\right)/T} \tag{20}$$

$$\gamma_x = 2.8950 \cdot 10^{10} T e^{\left(-23612+63.735T-18.206V_{m,x}\right)/T} \tag{21}$$

$$\delta_x = 2.8950 \cdot 10^{10} T e^{\left(-16078+27.565T-18.064V_{m,x}\right)/T} \tag{22}$$

$$c_n = 1.4475 \cdot 10^{9} T e^{\left(-7163.7/T+0.00086\right)} \tag{23}$$

$$c_f = 2.8950 \cdot 10^{8} T e^{\left(-34626.6/T+94.554\right)} \tag{24}$$

$$\varepsilon = 2.8950 \cdot 10^{10} T e^{\left(-10318.7/T+8.4279\right)} \tag{25}$$

$$\eta_x = 2.8950 \cdot 10^{10} T e^{\left(-17777+40.760T-8.6955V_{m,x}\right)/T} \tag{26}$$

$$\nu_x = 2.0842 \cdot 10^{10} T e^{\left(-8147.6+0.2127T-19.71V_{m,x}\right)/T} \tag{27}$$

$$\omega_x = \frac{\varepsilon \nu_x \gamma_x}{\eta_x \delta_x} \tag{28}$$

$$\delta\delta_x = 2.8950 \cdot 10^{10} T e^{\left(-6698.9-15.711T-42.316V_{m,x}\right)/T} \tag{29}$$

$$\gamma\gamma_x = 2.8950 \cdot 10^{10} T e^{\left(14003-69.55105T+8.8590V_{m,x}\right)/T} \tag{30}$$

$$o_{n,x} = 2.0842 \cdot 10^{10} T e^{\left(-7471.2+3.4498T+3.3483V_{m,x}\right)/T} \tag{31}$$

$$o_{f,x} = \frac{\delta\delta_x o_{n,x} \gamma_x c_f}{a^8 c_n \delta_x \gamma\gamma_x} \tag{32}$$

$$a = 7.5 \tag{33}$$

Persistent Na$^+$ Current, I_{Naps}

$$I_{Naps,x} = g_{Naps,x} k_{Naps,x} (V_{m,x} - E_{Naps,x}) \tag{34}$$

$$k_{Naps,x} = \frac{1}{1+e^{-(V_{m,x}+54)/8}} \tag{35}$$

$$E_{Naps,x} = \frac{RT}{F} ln\left(\frac{\left[Na^+\right]_o + 0.12\left[K^+\right]_o}{\left[Na^+\right]_i + 0.12\left[K^+\right]_i}\right) \tag{36}$$

L-type Ca^{2+} Current, I_{CaL}

$$I_{CaL,x}=P_{CaL,x}(O_x+O_{Ca,x})y_xV_{m,x}\frac{4F^2}{RT}\frac{0.001e^{2V_{m,x}F/(RT)}-0.341\left[Ca^{2+}\right]_o}{e^{2V_{m,x}F/(RT)}-1} \tag{37}$$

$$\frac{dC_{0,x}}{dt}=\beta_xC_{1,x}+\omega C_{Ca0,x}-(4\alpha_x+\gamma)C_{0,x} \tag{38}$$

$$\frac{dC_{1,x}}{dt}=4\alpha_xC_{0,x}-2\beta_xC_{2,x}+\frac{\omega}{b}C_{Ca1,x}-(\beta_x+3\alpha_x+\gamma a)C_{1,x} \tag{39}$$

$$\frac{dC_{2,x}}{dt}=3\alpha_xC_{1,x}+3\beta_xC_{3,x}+\frac{\omega}{b^2}C_{Ca2,x}-(2\beta_x+2\alpha_x+\gamma a^2)C_{2,x} \tag{40}$$

$$\frac{dC_{3,x}}{dt}=2\alpha_xC_{2,x}+4\beta_xC_{4,x}+\frac{\omega}{b^3}C_{Ca3,x}-(3\beta_x+\alpha_x+\gamma a^3)C_{3,x} \tag{41}$$

$$\frac{dC_{4,x}}{dt}=\alpha_xC_{3,x}+gO_x+\frac{\omega}{b^4}C_{Ca4,x}-(4\beta_x+f+\gamma a^4)C_{4,x} \tag{42}$$

$$\frac{dO_x}{dt}=fC_{4,x}-gO_x \tag{43}$$

$$\frac{dC_{Ca0,x}}{dt}=\beta'_xC_{Ca1,x}+\gamma C_{0,x}-(4\alpha'_x+\omega)C_{Ca0,x} \tag{44}$$

$$\frac{dC_{Ca1,x}}{dt}=4\alpha'_xC_{Ca0,x}+2\beta'_xC_{Ca2,x}+\gamma aC_{1,x}-(\beta'_x+3\alpha'_x+\frac{\omega}{b})C_{Ca1,x} \tag{45}$$

$$\frac{dC_{Ca2,x}}{dt}=3\alpha'_xC_{Ca1,x}+3\beta'_xC_{Ca3,x}+\gamma a^2C_{2,x}-(2\beta'_x+2\alpha'_x+\frac{\omega}{b^2})C_{Ca2,x} \tag{46}$$

$$\frac{dC_{Ca3,x}}{dt}=2\alpha'_xC_{Ca2,x}+4\beta'_xC_{Ca4,x}+\gamma a^3C_{3,x}-(3\beta'_x+\alpha'_x+\frac{\omega}{b^3})C_{Ca3,x} \tag{47}$$

$$\frac{dC_{Ca4,x}}{dt}=\alpha'_xC_{Ca3,x}+g'_xO_{Ca,x}+\gamma a^4C_{4,x}-(4\beta'_x+f'+\frac{\omega}{b^4})C_{Ca4,x} \tag{48}$$

$$\frac{dO_{Ca,x}}{dt}=f'C_{Ca4,x}-g'O_{Ca,x} \tag{49}$$

$$\alpha_x=1997e^{0.012(V_{m,x}-35)} \tag{50}$$

$$\beta_x=88.2e^{-0.065(V_{m,x}-22)} \tag{51}$$

$$\alpha'_x=a\alpha_x \tag{52}$$

$$\beta'_x=\frac{\beta_x}{b} \tag{53}$$

$$\gamma_t=55.4\left[Ca^{2+}\right]_{ss},\ \gamma_s=55.4\left[Ca^{2+}\right]_i \tag{54}$$

$$\frac{\mathrm{d}y_{\mathrm{x}}}{\mathrm{d}t}=\frac{\bar{y}_{\mathrm{x}}\text{-}y_{\mathrm{x}}}{\tau_{y_{\mathrm{x}}}} \tag{55}$$

$$\bar{y}_{\mathrm{x}}=\frac{0.82}{1+e^{(V_{\mathrm{m,x}}+28.5)/7.8}}+0.1 \tag{56}$$

$$\tau_{y_{\mathrm{x}}}=\frac{0.001}{\dfrac{0.00653}{0.5+e^{-V_{\mathrm{m,x}}/7.1}}+0.00512e^{-V_{\mathrm{m,x}}/39.8}} \tag{57}$$

L-Type K$^+$ Current, I$_{\mathbf{KL}}$

$$I_{\mathrm{KL,x}}=P'_{\mathrm{KL,x}}(O_{\mathrm{x}}+O_{\mathrm{Ca,x}})y_{\mathrm{x}}V_{\mathrm{m,x}}\frac{F^2}{RT}\frac{\left[\mathrm{K}^+\right]_{\mathrm{i}}e^{V_{\mathrm{m,x}}F/(RT)}-\left[\mathrm{K}^+\right]_{\mathrm{o}}}{e^{V_{\mathrm{m,x}}F/(RT)}-1} \tag{58}$$

$$P'_{\mathrm{KL,x}}=\frac{P_{\mathrm{KL,x}}}{1+\dfrac{I_{\mathrm{CaL}}}{I_{\mathrm{CaL,half}}}} \tag{59}$$

Transient Outward K$^+$ Current, I$_{\mathbf{Kto}}$

$$I_{\mathrm{Kto,x}}=g_{\mathrm{Kto,x}}r_{\mathrm{x}}s_{\mathrm{x}}(V_{\mathrm{m,x}}-E_{\mathrm{Kto,x}}) \tag{60}$$

$$\frac{\mathrm{d}r_{\mathrm{x}}}{\mathrm{d}t}=\frac{\bar{r}_{\mathrm{x}}-r_{\mathrm{x}}}{\tau_{r_{\mathrm{x}}}} \tag{61}$$

$$\frac{\mathrm{d}s_{\mathrm{x}}}{\mathrm{d}t}=\frac{\bar{s}_{\mathrm{x}}-s_{\mathrm{x}}}{\tau_{s_{\mathrm{x}}}} \tag{62}$$

$$\bar{r}_{\mathrm{x}}=\frac{1}{1+e^{(20\text{-}V_{\mathrm{m,x}})/6}} \tag{63}$$

$$\bar{s}_{\mathrm{x}}=\frac{1}{1+e^{(V_{\mathrm{m,x}}+20)/5}} \tag{64}$$

$$\tau_{r_{\mathrm{x}}}=0.0095e^{-\left(\mathrm{V_{m,x}}+40\right)^2/1800}+0.0008 \tag{65}$$

$$\tau_{s_{\mathrm{x}}}=0.085e^{-\left(\mathrm{V_{m,x}}+45\right)^2/320}+\frac{0.005}{1+e^{\left(\mathrm{V_{m,x}}\text{-}63\right)/5}}+0.003 \tag{66}$$

$$E_{\mathrm{Kto,x}}=\frac{RT}{F}ln\left(\frac{\left[\mathrm{K}^+\right]_{\mathrm{o}}}{\left[\mathrm{K}^+\right]_{\mathrm{i}}}\right) \tag{67}$$

Rapid Component of Delayed Rectifier K+ Current, I_{Kr}

$$I_{\mathrm{Kr,x}} = g_{\mathrm{Kr,x}} c_{\mathrm{Kr,x}} x_{\mathrm{r1,x}} x_{\mathrm{r2,x}} (V_{\mathrm{m,x}} - E_{\mathrm{Kr,x}}) \tag{68}$$

$$c_{\mathrm{Kr,x}} = \left(\left[\mathrm{K}^+\right]_{\mathrm{o}} / 5.4\right)^{0.5} \tag{69}$$

$$\frac{\mathrm{d}x_{\mathrm{r1,x}}}{\mathrm{d}t} = \frac{\overline{x}_{\mathrm{r1,x}} - x_{\mathrm{r1,x}}}{\tau_{x_{\mathrm{r1,x}}}} \tag{70}$$

$$\frac{\mathrm{d}x_{\mathrm{r2,x}}}{\mathrm{d}t} = \frac{\overline{x}_{\mathrm{r2,x}} - x_{\mathrm{r2,x}}}{\tau_{x_{\mathrm{r2,x}}}} \tag{71}$$

$$\overline{x}_{\mathrm{r1,x}} = \frac{1}{1+e^{-(V_{\mathrm{m,x}}+26)/7}} \tag{72}$$

$$\overline{x}_{\mathrm{r2,x}} = \frac{1}{1+e^{(V_{\mathrm{m,x}}+88)/24}} \tag{73}$$

$$\tau_{x_{\mathrm{r1,x}}} = \frac{0.45}{1+e^{-\left(V_{\mathrm{m,x}}+45\right)/10}} \cdot \frac{6}{1+e^{\left(V_{\mathrm{m,x}}+30\right)/11.5}} \tag{74}$$

$$\tau_{x_{\mathrm{r2,x}}} = \frac{0.003}{1+e^{-\left(V_{\mathrm{m,x}}+60\right)/20}} \cdot \frac{1.12}{1+e^{\left(V_{\mathrm{m,x}}-60\right)/20}} \tag{75}$$

$$E_{\mathrm{Kr,x}} = E_{\mathrm{Kto,x}} \tag{76}$$

Slow Component of Delayed Rectifier K+ Current, I_{Ks}

$$I_{\mathrm{Ks,x}} = g_{\mathrm{Ks,x}} x_{\mathrm{s,x}}^2 (V_{\mathrm{m,x}} - E_{\mathrm{Ks,x}}) \tag{77}$$

$$\frac{\mathrm{d}x_{\mathrm{s,x}}}{\mathrm{d}t} = \frac{\overline{x}_{\mathrm{s,x}} - x_{\mathrm{s,x}}}{\tau_{x_{\mathrm{s,x}}}} \tag{78}$$

$$\overline{x}_{\mathrm{s,x}} = \frac{1}{1+e^{-(V_{\mathrm{m,x}}-1.5)/14}} \tag{79}$$

$$\tau_{x_{\mathrm{s,x}}} = \frac{1.1}{\left(1+e^{-\left(V_{\mathrm{m,x}}+10\right)/6}\right)^{0.5}} \cdot \frac{1}{1+e^{\left(V_{\mathrm{m,x}}-60\right)/20}} \tag{80}$$

$$E_{\mathrm{Ks,x}} = \frac{RT}{F} ln\left(\frac{\left[\mathrm{K}^+\right]_{\mathrm{o}} + 0.03\left[\mathrm{Na}^+\right]_{\mathrm{o}}}{\left[\mathrm{K}^+\right]_{\mathrm{i}} + 0.03\left[\mathrm{Na}^+\right]_{\mathrm{i}}}\right) \tag{81}$$

Inward Rectifying K+ Current, I_{K1}

$$I_{\mathrm{K1,x}} = g_{\mathrm{K1,x}} c_{\mathrm{K1,x}} k_{\mathrm{K1,x}} (V_{\mathrm{m,x}} - E_{\mathrm{K1,x}}) \tag{82}$$

$$c_{\mathrm{K1,x}} = \left(\left[\mathrm{K}^+\right]_{\mathrm{o}}\right)^{0.5} \tag{83}$$

$$k_{\mathrm{K1,x}} = \frac{1}{0.94 + e^{1.26\left(V_{\mathrm{m,x}} - E_{\mathrm{K1,x}}\right)\cdot\left(F/RT\right)}} \tag{84}$$

$$E_{\mathrm{K1,x}} = E_{\mathrm{Kto,x}} \tag{85}$$

Na^+ Activated K^+ Current, $I_{K(Na)}$

$$I_{\mathrm{K(Na),x}} = g_{\mathrm{K(Na),x}} c_{\mathrm{K(Na)}}\ k_{\mathrm{K(Na),x}} (V_{\mathrm{m,x}} - E_{\mathrm{K(Na),x}}) \tag{86}$$

$$c_{\mathrm{K(Na)}} = \frac{0.85}{1 + \left(\dfrac{K_{\mathrm{half,K(Na_i)}}}{\left[\mathrm{Na}^+\right]_{\mathrm{i}}}\right)^{2.8}} \tag{87}$$

$$k_{\mathrm{K(Na),x}} = 0.8 - \frac{0.65}{1 + e^{(V_{\mathrm{m,x}} + 125)/15}} \tag{88}$$

$$E_{\mathrm{K(Na),x}} = E_{\mathrm{Kto,x}} \tag{89}$$

Non-Specific Ca^{2+} Activated Current, $I_{ns(Ca)}$

$$I_{\mathrm{ns(Ca),x}} = I_{\mathrm{nsNa,x}} + I_{\mathrm{nsK,x}} \tag{90}$$

$$I_{\mathrm{nsNa,x}} = P_{\mathrm{nsNa,x}} c_{\mathrm{nsNa}} V_{\mathrm{m,x}} \frac{F^2}{RT} \frac{0.75\left[\mathrm{Na}^+\right]_{\mathrm{i}} e^{V_{\mathrm{m,x}}F/(RT)} - 0.75\left[\mathrm{Na}^+\right]_{\mathrm{o}}}{e^{V_{\mathrm{m,x}}F/(RT)} - 1} \tag{91}$$

$$I_{\mathrm{nsK,x}} = P_{\mathrm{nsK,x}} c_{\mathrm{nsK}} V_{\mathrm{m,x}} \frac{F^2}{RT} \frac{0.75\left[\mathrm{K}^+\right]_{\mathrm{i}} e^{V_{\mathrm{m,x}}F/(RT)} - 0.75\left[\mathrm{K}^+\right]_{\mathrm{o}}}{e^{V_{\mathrm{m,x}}F/(RT)} - 1} \tag{92}$$

$$c_{\mathrm{nsNa}} = \frac{1}{1 + \left(\dfrac{K_{\mathrm{half,nsNa(Ca_i)}}}{\left[\mathrm{Ca}^{2+}\right]_{\mathrm{i}}}\right)^{3}},\ c_{\mathrm{nsK}} = \frac{1}{1 + \left(\dfrac{K_{\mathrm{half,nsK(Ca_i)}}}{\left[\mathrm{Ca}^{2+}\right]_{\mathrm{i}}}\right)^{3}} \tag{93}$$

Background Current, I_b

$$I_{\mathrm{b,x}} = I_{\mathrm{Nab,x}} + I_{\mathrm{Cab,x}} \tag{94}$$

$$I_{\mathrm{Nab,x}} = g_{\mathrm{Nab,x}} (V_{\mathrm{m,x}} - E_{\mathrm{Na,x}}) \tag{95}$$

$$I_{\mathrm{Cab,x}} = g_{\mathrm{Cab,x}} (V_{\mathrm{m,x}} - E_{\mathrm{Ca,x}}) \tag{96}$$

$$E_{\mathrm{Nab,x}} = \frac{RT}{F} ln\left(\frac{\left[\mathrm{Na}^+\right]_{\mathrm{o}}}{\left[\mathrm{Na}^+\right]_{\mathrm{i}}}\right) \tag{97}$$

$$E_{\mathrm{Cab,x}} = \frac{RT}{2F} ln\left(\frac{\left[\mathrm{Ca}^+\right]_{\mathrm{o}}}{\left[\mathrm{Ca}^+\right]_{\mathrm{i}}}\right) \tag{98}$$

Na^{+}- Ca^{2+} Exchanger Current, I_{NaCa}

$$I_{\mathrm{NaCa,x}} = k_{\mathrm{NaCa,x}} e^{-0.85 V_{\mathrm{m,x}} F/(RT)} \times \frac{e^{V_{\mathrm{m,x}} F/(RT)} \left[\mathrm{Na}^{+}\right]_{\mathrm{i}}^{3} \left[\mathrm{Ca}^{+}\right]_{\mathrm{o}} - \left[\mathrm{Na}^{+}\right]_{\mathrm{o}}^{3} \left[\mathrm{Ca}^{+}\right]_{\mathrm{i}}}{1+0.0001 e^{-0.85 V_{\mathrm{m,x}} F/(RT)} \left(\mathrm{e}^{V_{\mathrm{m,x}} F/(RT)} \left[\mathrm{Na}^{+}\right]_{\mathrm{i}}^{3} \left[\mathrm{Ca}^{+}\right]_{\mathrm{o}} + \left[\mathrm{Na}^{+}\right]_{\mathrm{o}}^{3} \left[\mathrm{Ca}^{+}\right]_{\mathrm{i}} \right)} \tag{99}$$

Na^{+}- K^{+} Pump Current, I_{NaK}

$$I_{\mathrm{NaK,x}} = I_{\mathrm{NaK,max,x}} c1_{\mathrm{NaK,x}} c2_{\mathrm{NaK}} k_{\mathrm{NaK,x}} \tag{100}$$

$$c1_{\mathrm{NaK,x}} = \frac{\left[\mathrm{K}^{+}\right]_{\mathrm{o}}}{\left[\mathrm{K}^{+}\right]_{\mathrm{o}} + K_{\mathrm{half,NaK(Ko)}}} \tag{101}$$

$$c2_{\mathrm{NaK}} = \frac{1}{1+\left(\frac{K_{\mathrm{half,NaK(Na_i)}}}{\left[\mathrm{Na}^{+}\right]_{\mathrm{i}}} \right)^{1.5}} \tag{102}$$

$$k_{\mathrm{NaK,x}} = \frac{1}{1+0.1245 e^{-0.1 V_{\mathrm{m,x}} \mathrm{F/(RT)}} + 0.0365 \sigma_{\mathrm{x}} e^{-V_{\mathrm{m,x}} \mathrm{F/(RT)}}} \tag{103}$$

$$\sigma_{\mathrm{x}} = \frac{1}{7} \left(e^{\left[\mathrm{Na}^{+}\right]_{\mathrm{o}}/67.3} - 1 \right) \tag{104}$$

Ca^{2+} Pump Current, I_{pCa}

$$I_{\mathrm{pCa,x}} = I_{\mathrm{pCa,max,x}} c_{\mathrm{pCa}} \tag{105}$$

$$c_{\mathrm{pCa}} = \frac{\left[\mathrm{Ca}^{2+}\right]_{\mathrm{i}}}{K_{\mathrm{half,pCa(Ca_i)}} + \left[\mathrm{Ca}^{2+}\right]_{\mathrm{i}}} \tag{106}$$

Circulation Current, I_{circ}

$$I_{\mathrm{circ}} = \left[\frac{V_{\mathrm{ms}} - V_{\mathrm{mt}}}{R_{\mathrm{st}}} \right] \tag{107}$$

Stimulation Current, I_{stim}

$$I_{\mathrm{stim}} = -45 \text{ mA cm}^{-2} \text{ for } 0.001 \text{ s} \tag{108}$$

Intracellular Ca^{2+} Handling

The values of parameters and constants used in the description of intracellular Ca^{2+} handling are listed in Tables S6 and S7.

Ca^{2+} Uptake From Cytoplasm Into NSR, J_{SRup}

$$J_{SRup} = K_{SR}\left(\frac{v_{maxf}\, f_b - v_{maxr} r_b}{1+f_b+r_b}\right) \tag{109}$$

$$f_b = \left(\frac{\left[Ca^{2+}\right]_i}{K_{fb}}\right)^{N_{fb}} \tag{110}$$

$$r_b = \left(\frac{\left[Ca^{2+}\right]_{NSR}}{K_{rb}}\right)^{N_{rb}} \tag{111}$$

Ca^{2+} Transfer from NSR into JSR, J_{SRtr}

$$J_{SRtr} = \frac{\left[Ca^{2+}\right]_{NSR} - \left[Ca^{2+}\right]_{JSR}}{\tau_{tr}} \tag{112}$$

Ca^{2+} Release from JSR into Subspace, J_{SRrel}

$$J_{SRrel} = \left(P_{O1}+P_{O2}\right)\frac{\left[Ca^{2+}\right]_{JSR} - \left[Ca^{2+}\right]_{ss}}{\tau_{rel}} \tag{113}$$

$$\frac{dP_{C1}}{dt} = P_{O1}k_a^- - P_{C1}k_a^+\left[Ca^{2+}\right]_{ss}^4 \tag{114}$$

$$\frac{dP_{O1}}{dt} = P_{C1}k_a^+\left[Ca^{2+}\right]_{ss}^4 + P_{O2}k_b^- + P_{C2}k_c^- - P_{O1}(k_a^- + k_b^+\left[Ca^{2+}\right]_{ss}^3 + k_c^+) \tag{115}$$

$$\frac{dP_{O2}}{dt} = P_{O1}k_b^+\left[Ca^{2+}\right]_{ss}^3 - P_{O2}k_b^- \tag{116}$$

$$\frac{dP_{C2}}{dt} = P_{O1}k_c^+ - P_{C2}k_c^- \tag{117}$$

Ca^{2+} Transfer from Subspace into Cytoplasm, J_{cyt}

$$J_{cyt} = \frac{\left[Ca^{2+}\right]_{ss} - \left[Ca^{2+}\right]_i}{\tau_{xfer}} \tag{118}$$

Ca^{2+} Buffering by Troponin, J_{trpn}

$$J_{trpn} = V_{myo}\left(\frac{d\left[HTRPN_{Ca}\right]}{dt} + \frac{d\left[LTRPN_{Ca}\right]}{dt}\right) \tag{119}$$

$$\frac{d\left[HTRPN_{Ca}\right]}{dt} = k_{htrpn}^+\left[Ca^{2+}\right]_i\left(\left[HTRPN_{Ca}\right]_{tot} - \left[HTRPN_{Ca}\right]\right) - k_{htrpn}^-\left[HTRPN_{Ca}\right] \tag{120}$$

$$\frac{\mathrm{d}\left[\mathrm{LTRPN_{Ca}}\right]}{\mathrm{d}t}=k^{+}_{\mathrm{ltrpn}}\left[\mathrm{Ca}^{2+}\right]_{\mathrm{i}}\left(\left[\mathrm{LTRPN_{Ca}}\right]_{\mathrm{tot}}-\left[\mathrm{LTRPN_{Ca}}\right]\right)-k^{-}_{\mathrm{ltrpn}}\left[\mathrm{LTRPN_{Ca}}\right] \tag{121}$$

Ca^{2+} Buffering by Calmodulin, B_i and B_{ss}

$$B_{\mathrm{i}}=\left\{1+\frac{\left[\mathrm{CMDN_{Ca}}\right]_{\mathrm{tot}}K_{\mathrm{half,CMDN(Ca)}}}{\left(K_{\mathrm{half,CMDN(Ca)}}+\left[\mathrm{Ca}^{2+}\right]_{\mathrm{i}}\right)^{2}}\right\}^{-1} \tag{122}$$

$$B_{\mathrm{ss}}=\left\{1+\frac{\left[\mathrm{CMDN_{Ca}}\right]_{\mathrm{tot}}K_{\mathrm{half,CMDN(Ca)}}}{\left(K_{\mathrm{half,CMDN(Ca)}}+\left[\mathrm{Ca}^{2+}\right]_{\mathrm{ss}}\right)^{2}}\right\}^{-1} \tag{123}$$

Ca^{2+} Buffering by Calsequestrin, B_{JSR}

$$B_{\mathrm{JSR}}=\left\{1+\frac{\left[\mathrm{CSQN_{Ca}}\right]_{\mathrm{tot}}K_{\mathrm{half,CSQN(Ca)}}}{\left(K_{\mathrm{half,CSQN(Ca)}}+\left[\mathrm{Ca}^{2+}\right]_{\mathrm{JSR}}\right)^{2}}\right\}^{-1} \tag{124}$$

Ion Concentrations

The time constants of ion exchange between individual tubular segments, between cleft and t-tubular spaces and between external bulk and cleft spaces are specified in Tables S8–S10.

Intracellular Ion Concentrations

$$\frac{\mathrm{d}\left[\mathrm{Na}^{+}\right]_{\mathrm{i}}}{\mathrm{d}t}=-\left(\frac{I_{\mathrm{Na,s}}+I_{\mathrm{Na,t}}+I_{\mathrm{Naps,s}}+I_{\mathrm{Naps,t}}+I_{\mathrm{nsNa,s}}+I_{\mathrm{nsNa,t}}+I_{\mathrm{Nab,s}}+I_{\mathrm{Nab,t}}+3I_{\mathrm{NaCa,s}}+3I_{\mathrm{NaCa,t}}}{FV_{\mathrm{myo}}}\right)-\left(\frac{3I_{\mathrm{NaK,s}}+3I_{\mathrm{NaK,t}}}{FV_{\mathrm{myo}}}\right) \tag{125}$$

$$\frac{\mathrm{d}\left[\mathrm{K}^{+}\right]_{\mathrm{i}}}{\mathrm{d}t}=-\left(\frac{I_{\mathrm{Kto,s}}+I_{\mathrm{Kto,t}}+I_{\mathrm{Kr,s}}+I_{\mathrm{Kr,t}}+I_{\mathrm{Ks,s}}+I_{\mathrm{Ks,t}}+I_{\mathrm{K1,s}}+I_{\mathrm{K1,t}}+I_{\mathrm{KL,s}}+I_{\mathrm{KL,t}}+I_{\mathrm{K(Na),s}}+I_{\mathrm{K(Na),t}}}{FV_{\mathrm{myo}}}\right)-\left(\frac{I_{\mathrm{nsK,s}}+I_{\mathrm{nsK,t}}-2I_{\mathrm{NaK,s}}-2I_{\mathrm{NaK,t}}+I_{\mathrm{stim}}}{FV_{\mathrm{myo}}}\right) \tag{126}$$

$$\frac{\mathrm{d}\left[\mathrm{Ca}^{2+}\right]_{\mathrm{i}}}{\mathrm{d}t}=B_{\mathrm{i}}\left(\frac{2I_{\mathrm{NaCa,s}}+2I_{\mathrm{NaCa,t}}-I_{\mathrm{CaL,s}}-I_{\mathrm{Cab,s}}-I_{\mathrm{Cab,t}}-I_{\mathrm{pCa,s}}-I_{\mathrm{pCa,t}}}{2FV_{\mathrm{myo}}}+\frac{J_{\mathrm{cyt}}-J_{\mathrm{SRup}}-J_{\mathrm{trpn}}}{V_{\mathrm{myo}}}\right) \tag{127}$$

$$\frac{\mathrm{d}\left[\mathrm{Ca}^{2+}\right]_{\mathrm{ss}}}{\mathrm{d}t}=B_{\mathrm{ss}}\left(\frac{-I_{\mathrm{CaL,t}}}{2FV_{\mathrm{ss}}}+\frac{J_{\mathrm{SRrel}}-J_{\mathrm{cyt}}}{V_{\mathrm{ss}}}\right) \tag{128}$$

$$\frac{\mathrm{d}\left[\mathrm{Ca}^{2+}\right]_{\mathrm{NSR}}}{\mathrm{d}t}=\frac{J_{\mathrm{SRup}}-J_{\mathrm{SRtr}}}{V_{\mathrm{NSR}}} \quad (129)$$

$$\frac{\mathrm{d}\left[\mathrm{Ca}^{2+}\right]_{\mathrm{JSR}}}{\mathrm{d}t}=B_{\mathrm{JSR}}\frac{J_{\mathrm{SRtr}}-J_{\mathrm{SRrel}}}{V_{\mathrm{JSR}}} \quad (130)$$

Extracellular Ion Concentrations

$$\frac{\mathrm{d}\left[\mathrm{Na}^{+}\right]_{\mathrm{t}}}{\mathrm{d}t}=\frac{I_{\mathrm{Na,t}}+I_{\mathrm{Naps,t}}+I_{\mathrm{nsNa,t}}+I_{\mathrm{Nab,t}}+3I_{\mathrm{NaCa,t}}+3I_{\mathrm{NaK,t}}}{FV_{\mathrm{t}}}+\frac{J_{\mathrm{Na,ct}}}{V_{\mathrm{t}}} \quad (131)$$

$$J_{\mathrm{Na,ct}}=\frac{V_{\mathrm{t}}}{\tau_{\mathrm{Na,ct}}}\left(\left[\mathrm{Na}^{+}\right]_{\mathrm{c}}-\left[\mathrm{Na}^{+}\right]_{\mathrm{t}}\right) \quad (132)$$

$$\frac{\mathrm{d}\left[\mathrm{K}^{+}\right]_{\mathrm{St,1}}}{\mathrm{d}t}=\frac{I_{\mathrm{Kto,t}}+I_{\mathrm{Kr,t}}+I_{\mathrm{Ks,t}}+I_{\mathrm{K1,t}}+I_{\mathrm{KL,t}}+I_{\mathrm{K(Na),t}}+I_{\mathrm{nsK,t}}-2I_{\mathrm{NaK,t}}}{FV_{\mathrm{St,1}}}+\frac{J_{\mathrm{K,ct,1}}-J_{\mathrm{K,St,2}}}{V_{\mathrm{St,1}}} \quad (133)$$

$$\frac{\mathrm{d}\left[\mathrm{K}^{+}\right]_{\mathrm{St,n}}}{\mathrm{dt}}=\frac{\mathrm{J}_{\mathrm{K,ct,n}}+\mathrm{J}_{\mathrm{K,St,n}}-\mathrm{J}_{\mathrm{K,St,n+1}}}{\mathrm{V}_{\mathrm{St,n}}},\quad n=(2,3,...9) \quad (134)$$

$$J_{\mathrm{K,ct,n}}=\frac{V_{\mathrm{St,n}}}{\tau_{\mathrm{K,ct}}}\left(\left[\mathrm{K}^{+}\right]_{\mathrm{c}}-\left[\mathrm{K}^{+}\right]_{\mathrm{St,n}}\right),\quad n=(1,2,...9) \quad (135)$$

$$J_{\mathrm{K,St,n+1}}=\frac{V_{\mathrm{St,n+1}}}{\tau_{\mathrm{K,St,n+1}}}\left(\left[\mathrm{K}^{+}\right]_{\mathrm{St,n}}-\left[\mathrm{K}^{+}\right]_{\mathrm{St,n+1}}\right),\quad n=(1,2,..8) \quad (136)$$

$$\frac{\mathrm{d}\left[\mathrm{Ca}^{2+}\right]_{\mathrm{St,1}}}{\mathrm{d}t}=\frac{I_{\mathrm{Ca,t}}+I_{\mathrm{Cab,t}}+I_{\mathrm{pCa,t}}-2I_{\mathrm{NaCa,t}}}{2FV_{\mathrm{St,1}}}+\frac{J_{\mathrm{Ca,ct,1}}-J_{\mathrm{Ca,St,2}}}{V_{\mathrm{St,1}}} \quad (137)$$

$$\frac{\mathrm{d}\left[Ca^{2+}\right]_{\mathrm{St,n}}}{\mathrm{dt}}=\frac{\mathrm{J}_{\mathrm{Ca,ct,n}}+\mathrm{J}_{\mathrm{Ca,St,n}}-\mathrm{J}_{\mathrm{Ca,St,n+1}}}{\mathrm{V}_{\mathrm{St,n}}},\quad n=(2,3,...9) \quad (138)$$

$$J_{\mathrm{Ca,ct,n}}=\frac{V_{\mathrm{St,n}}}{\tau_{\mathrm{Ca,ct}}}\left(\left[\mathrm{Ca}^{2+}\right]_{\mathrm{c}}-\left[\mathrm{Ca}^{2+}\right]_{\mathrm{St,n}}\right),\quad n=(1,2,...9) \quad (139)$$

$$J_{\mathrm{Ca,St,n+1}}=\frac{V_{\mathrm{St,n+1}}}{\tau_{\mathrm{Ca,St,n+1}}}\left(\left[Ca^{2+}\right]_{\mathrm{St,n}}-\left[Ca^{2+}\right]_{\mathrm{St,n+1}}\right),\quad n=(1,2,..8) \quad (140)$$

$$\frac{\mathrm{d}\left[\mathrm{Na}^{+}\right]_{\mathrm{c}}}{\mathrm{d}t}=\frac{I_{\mathrm{Na,s}}+I_{\mathrm{Naps,s}}+I_{\mathrm{nsNa,t}}+I_{\mathrm{Nab,s}}+3I_{\mathrm{NaCa,s}}+3I_{\mathrm{NaK,s}}}{FV_{\mathrm{e}}}-\frac{J_{\mathrm{Na,ct}}}{V_{\mathrm{e}}}+\frac{1}{\tau_{\mathrm{Na,bc}}}\left(\left[Na^{+}\right]_{\mathrm{b}}-\left[Na^{+}\right]_{\mathrm{c}}\right) \quad (141)$$

$$\frac{\mathrm{d}\left[\mathrm{Ca}^{2+}\right]_{\mathrm{c}}}{\mathrm{d}t}=\frac{I_{\mathrm{Ca,s}}+I_{\mathrm{Cab,s}}+I_{\mathrm{pCa,s}}-2I_{\mathrm{NaCa,s}}}{2FV_{\mathrm{e}}}-\frac{\sum_{\mathrm{n}}J_{\mathrm{Ca,ct,n}}}{V_{\mathrm{e}}}+\frac{1}{\tau_{\mathrm{Ca,bc}}}\left(\left[\mathrm{Ca}^{2+}\right]_{\mathrm{b}}-\left[\mathrm{Ca}^{2+}\right]_{\mathrm{c}}\right) \quad (142)$$

$$\frac{\mathrm{d}\left[\mathrm{K}^+\right]_\mathrm{c}}{\mathrm{d}t}=\frac{I_{\mathrm{Kto,s}}+I_{\mathrm{Kr,s}}+I_{\mathrm{Ks,s}}+I_{\mathrm{K1,s}}+I_{\mathrm{KL,s}}+I_{\mathrm{K(Na),s}}+I_{\mathrm{nsK,s}}-2I_{\mathrm{NaK,s}}+0.001I_{\mathrm{stim}}(S_{\mathrm{m,s}}+S_{\mathrm{m,t}})/t_{\mathrm{cycle}}}{FV_\mathrm{e}} -\frac{\sum_\mathrm{n} J_{\mathrm{K,ct,n}}}{V_\mathrm{e}}+\frac{1}{\tau_{\mathrm{K,bc}}}\left(\left[\mathrm{K}^+\right]_\mathrm{b}-\left[\mathrm{K}^+\right]_\mathrm{c}\right),\ t_{\mathrm{cycle}}=\text{stimulation period} \quad (143)$$

Membrane Voltage

Surface Membrane Voltage

$$\frac{\mathrm{d}V_{\mathrm{m,s}}}{\mathrm{d}t}=\frac{I_{\mathrm{circ}}-I_{\mathrm{Na,s}}-I_{\mathrm{Naps,s}}-I_{\mathrm{CaL,s}}-I_{\mathrm{KL,s}}-I_{\mathrm{Kto,s}}-I_{\mathrm{Kr,s}}-I_{\mathrm{Ks,s}}-I_{\mathrm{K1,s}}-I_{\mathrm{K(Na),s}}-I_{\mathrm{ns(Ca),s}}-I_{\mathrm{Nab,s}}-I_{\mathrm{Cab,s}}}{C_{\mathrm{m,s}}}+\frac{-I_{\mathrm{NaCa,s}}-I_{\mathrm{NaK,s}}-I_{\mathrm{pCa,s}}-I_{\mathrm{stim}}}{C_{\mathrm{m,s}}} \quad (144)$$

Tubular Membrane Voltage

$$\frac{\mathrm{d}V_{\mathrm{m,t}}}{\mathrm{d}t}=\frac{-I_{\mathrm{circ}}-I_{\mathrm{Na,t}}-I_{\mathrm{Naps,t}}-I_{\mathrm{CaL,t}}-I_{\mathrm{KL,t}}-I_{\mathrm{Kto,t}}-I_{\mathrm{Kr,t}}-I_{\mathrm{Ks,t}}-I_{\mathrm{K1,t}}-I_{\mathrm{K(Na),t}}-I_{\mathrm{ns(Ca),t}}-I_{\mathrm{Nab,t}}-I_{\mathrm{Cab,t}}}{C_{\mathrm{m,t}}}+\frac{-I_{\mathrm{NaCa,t}}-I_{\mathrm{NaK,t}}-I_{\mathrm{pCa,t}}}{C_{\mathrm{m,t}}} \quad (145)$$

Table S2. Physical constants.

Parameter	Definition	Value
ρ_{ext}	Specific resistance of extracellular solution	83.33 Ωcm
C_{sc}	Specific capacitance of cellular membrane	0.01 pF μm^{-2}
R	Gas molar constant	8.314 J K^{-1} mol^{-1}
F	Faraday constant	96485 A s mol^{-1}
T	Absolute temperature	310 K

Table S3. Geometrical parameters.

Parameter	Definition	Value
L_c	Length of cell	130 μm
L_t	Mean length of tubules	5.015 μm
r_t	Mean radius of tubules	0.21 μm
ρ_t	Density of tubules in surface membrane	0.19 tubules μm^{-2}
$S_{m,s}$	Surface membrane area	6645 μm^2
$S_{m,t}$	Tubular membrane area	8355μm^2
V_c	Volume of cell	2.089 × 10^{-8} mL
V_{myo}	Volume of myoplasm	1.420 × 10^{-8} mL
V_{NSR}	Volume of network SR	0.115 × 10^{-8} mL
V_{JSR}	Volume of junctional SR	0.088 × 10^{-9} mL

Table S3. *Cont.*

Parameter	Definition	Value
V_{ss}	Volume of subspace	0.422×10^{-12} mL
V_t	Volume of TATS	8.772×10^{-10} mL
V_e	Volume of extracellular cleft space	1.9317×10^{-9} mL

Table S4. Membrane current parameters.

Parameter	Definition	Value
$K_{half,K(Nai)}$	$[Na^+]_i$ half saturation constant for $I_{K(Na)}$	66 mM
$K_{half,nsK(Cai)}$	$[Ca^{2+}]_i$ half saturation constant for I_{nsK}	0.0025 mM
$K_{half,nsNa(Cai)}$	$[Ca^{2+}]_i$ half saturation constant for I_{nsNa}	0.0025 mM
$K_{half,NaK(Ko)}$	$[K^+]_o$ half saturation constant for I_{NaK}	1.5 mM
$K_{half,NaK(Nai)}$	$[Na^+]_c$ half saturation constant for I_{NaK}	10 mM
$K_{half,pCa(Cai)}$	$[Ca^+]_i$ half saturation constant for I_{pCa}	0.0005 mM

Table S5. L-type Ca^{2+} channel parameters.

Parameter	Definition	Value
f	Transition rate into open state	300 s^{-1}
g	Transition rate out of open state	4000 s^{-1}
f'	Transition rate into open state for mode Ca	5 s^{-1}
g'	Transition rate out of open state for mode Ca	7000 s^{-1}
a	Mode transition parameter	2
b	Mode transition parameter	2
w	Mode transition parameter	10 s^{-1}
$I_{CaL,half}$	I_{CaL} level that reduces P_{KL} by half	−0.265 pA/pF

Table S6. SR parameters.

Parameter	Definition	Value
ν_{maxf}	Ca^{2+}-ATPase forward rate parameter	0.25 mM s^{-1}
ν_{maxr}	Ca^{2+}-ATPase reverse rate parameter	0.75 mM s^{-1}
K_{fb}	Ca^{2+}-ATPase forward half-saturation constant	0.000168 mM
K_{rb}	Ca^{2+}-ATPase backward half-saturation constant	3.29 mM
N_{fb}	Ca^{2+}-ATPase forward cooperativity constant	1.2
N_{rb}	Ca^{2+}-ATPase reverse cooperativity constant	1
K_{SR}	Ca^{2+}-ATPase scaling factor	1.2
τ_{tr}	τ of Ca^{2+} transfer from NSR to JSR	20 ms
τ_{rel}	τ of Ca^{2+} transfer from JSR to subspace	0.56 ms
τ_{xfer}	τ of Ca^{2+} transfer from subspace to cytosol	18 ms
k_a^+	RyR $P_{c1} – P_{o1}$ rate constant	12.5×10^{12} mM^{-4} s^{-1}
k_a^-	RyR $P_{o1} – P_{c1}$ rate constant	576 s^{-1}
k_b^+	RyR $P_{o1} – P_{o2}$ rate constant	4.05×10^9 mM^{-3} s^{-1}
k_b^-	RyR $P_{o2} – P_{o1}$ rate constant	1930 s^{-1}
k_c^+	RyR $P_{o1} – P_{c2}$ rate constant	100 s^{-1}
k_c^-	RyR $P_{c2} – P_{o1}$ rate constant	0.8 s^{-1}

Table S7. Buffering parameters.

Parameter	Definition	Value
$[HTRPN_{Ca}]_{tot}$	Total troponin high-affinity site concentration	0.14 mM
$[LTRPN_{Ca}]_{tot}$	Total troponin low-affinity site concentration	0.07 mM
$[CMDN_{Ca}]_{tot}$	Total calmodulin concentration in myoplasma	0.05 mM
$[CSQN_{Ca}]_{tot}$	Total calsequestrin concentration in JSR	15 mM
k^{+}_{htrpn}	Ca^{2+} on rate for troponin high-affinity sites	2×10^{4} mM^{-1} s^{-1}
k^{-}_{htrpn}	Ca^{2+} off rate for troponin high-affinity sites	0.07 s^{-1}
k^{+}_{ltrpn}	Ca^{2+} on rate for troponin low-affinity sites	4×10^{4} mM^{-1} s^{-1}
k^{-}_{ltrpn}	Ca^{2+} off rate for troponin low-affinity sites	40 s^{-1}
$K_{half,CMDN(Ca)}$	$[Ca^{2+}]_i$ half saturation constant for calmodulin	0.00238 mM
$K_{half,CSQN(Ca)}$	$[Ca^{2+}]_i$ half saturation constant for calsequestrin	0.8 mM

Table S8. Time constants of ion exchange between individual t-tubular segments.

Parameter	Definition	Value
$\tau_{K,St,2}$	τ of K^{+} exchange between t-tubular segments 1 and 2	1.276×10^{-4} s
$\tau_{K,St,3}$	τ of K^{+} exchange between t-tubular segments 2 and 3	1.296×10^{-4} s
$\tau_{K,St,4}$	τ of K^{+} exchange between t-tubular segments 3 and 4	1.317×10^{-4} s
$\tau_{K,St,5}$	τ of K^{+} exchange between t-tubular segments 4 and 5	1.339×10^{-4} s
$\tau_{K,St,6}$	τ of K^{+} exchange between t-tubular segments 5 and 6	1.363×10^{-4} s
$\tau_{K,St,7}$	τ of K^{+} exchange between t-tubular segments 6 and 7	1.387×10^{-4} s
$\tau_{K,St,8}$	τ of K^{+} exchange between t-tubular segments 7 and 8	1.413×10^{-4} s
$\tau_{K,St,9}$	τ of K^{+} exchange between t-tubular segments 8 and 9	3.651×10^{-3} s
$\tau_{Ca,St,2}$	τ of Ca^{2+}exchange between t-tubular segments 1 and 2	1.531×10^{-4} s
$\tau_{Ca,St,3}$	τ of Ca^{2+}exchange between t-tubular segments 2 and 3	1.555×10^{-4} s
$\tau_{Ca,St,4}$	τ of Ca^{2+}exchange between t-tubular segments 3 and 4	1.580×10^{-4} s
$\tau_{Ca,St,5}$	τ of Ca^{2+}exchange between t-tubular segments 4 and 5	1.607×10^{-4} s
$\tau_{Ca,St,6}$	τ of Ca^{2+}exchange between t-tubular segments 5 and 6	1.635×10^{-4} s
$\tau_{Ca,St,7}$	τ of Ca^{2+}exchange between t-tubular segments 6 and 7	1.665×10^{-4} s
$\tau_{Ca,St,8}$	τ of Ca^{2+}exchange between t-tubular segments 7 and 8	1.696×10^{-4} s
$\tau_{Ca,St,9}$	τ of Ca^{2+}exchange between t-tubular segments 8 and 9	4.381×10^{-3} s

Table S9. Time constants of ion exchange between cleft and t-tubular spaces.

Parameter	Definition	Value
$\tau_{Na,ct}$	τ of Na^{+} exchange between cleft and t-tubular spaces TATS	0.2 s
$\tau_{K,ct}$	τ of K^{+} exchange between cleft and t-tubular spaces	0.2 s
$\tau_{Ca,ct}$	τ of Ca^{2+} exchange between cleft and t-tubular spaces	0.24 s

Table S10. Time constants of ion exchange between external bulk and cleft spaces.

Parameter	Definition	Value
$\tau_{Na,bc}$	τ of Na^{+} exchange between external bulk and cleft spaces	14.3 s
$\tau_{K,bc}$	τ of K^{+} exchange between external bulk and cleft spaces	10 s
$\tau_{Ca,bc}$	τ of Ca^{2+} exchange between external bulk and cleft spaces	24.7 s
